# Hydrogen‐Deuterium Exchange Mass Spectrometry for Molecular Glue Characterization

**DOI:** 10.1002/advs.202508543

**Published:** 2025-08-04

**Authors:** Danielle F. Kay, Hadeeqa G. Raza, Richard G. Doveston, Aneika C. Leney

**Affiliations:** ^1^ School of Biosciences University of Birmingham Birmingham B15 2TT UK; ^2^ Institute of Structural and Chemical Biology and School of Chemistry University of Leicester Leicester LE1 7RH UK

**Keywords:** molecular glues, protein‐protein interactions, stabilization mechanisms, structural proteomics, ternary complexes

## Abstract

Molecular glues are powerful bioactive molecules that stabilize protein–protein interactions. Yet, the precise mechanisms by which many molecular glues exert their adhesive effects are still not well understood. Native mass spectrometry is an established technique used to monitor the stoichiometry and binding equilibria of molecular glue‐induced protein–protein interactions. However, knowledge is lacking on how protein interaction dynamics change upon molecular glue‐induced stabilization, and what conformational changes occur that enhance the stability of the resulting protein‐protein‐glue ternary complex. Here, hydrogen‐deuterium exchange mass spectrometry (HDX‐MS) is showcased as an analytical tool for molecular glue characterization. Using a broadly applicable molecular glue system involving the eukaryotic regulatory protein 14‐3‐3, its binding partners, and the molecular glue fusicoccin A, the power of HDX‐MS is shown in revealing not only molecular glue binding sites, but also conformational changes upon glue binding that result in differentially stabilized protein‐protein complexes. Overall, the HDX‐MS approach will become highly envisaged and valuable for the characterization of molecular glues, guiding their optimization toward successful design.

## Graphical abstract

Molecular glues that stabilize protein–protein interactions are challenging to identify. Here, hydrogen‐deuterium exchange mass spectrometry can show and reveal gluing mechanisms that vary between protein binding partners.

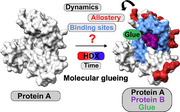

## 1 Introduction

Protein‐protein interactions (PPIs) are vital for every major cellular process, regulating cell signalling pathways, cell division, metabolism and the assembly of cellular machinery. Perturbing PPIs alters cellular homeostasis through consequential changes to downstream signalling mechanisms and intracellular processes. Moreover, the disruption of PPIs has been linked to a number of human diseases.^[^ ^1^, ^2^, ^3^ ^]^ As such, using molecular tools to enhance and stabilize these aberrant PPIs is a powerful therapeutic approach.^[^ ^4^ ^–^ ^6^ ^]^

Molecular glues (MGs) are a class of PPI modulator that induce or enhance the interaction between two proteins. This is achieved through complementary binding at a protein‐protein interface to form a stabilized ternary complex.^[^ ^7^, ^8^ ^]^ MGs typically only have affinity for a pre‐formed binary protein‐protein complex, but may show affinity for one protein partner. This distinguishes them from bifunctional molecules (e.g., proteolysis targeting chimeras, PROTACs) that combine high affinity ligands for both interacting proteins using a chemical linker. MGs can, therefore, be harnessed to target otherwise undruggable proteins to induce degradation,^[^ ^9^ ^]^ alter stability,^[^ ^10^ ^]^ inhibit,^[^ ^11^ ^]^ or activate^[^ ^12^ ^]^ protein function. The requirement for complementary molecular recognition of an interface between two proteins provides a platform for selectivity and avoids the “hook effect” often observed for bifunctional molecules.^[^ ^7^ ^]^ In addition, the absence of need for a molecular linker conveys significant advantages in terms of physiochemical properties and bioavailability. However, the discovery and optimization of such MGs is highly challenging: most are discovered serendipitiously,^[^ ^7^, ^13^ ^]^ with two classic examples being thalidomide^[^ ^14^ ^]^ and rapamycin.^[^ ^15^ ^]^

Screening for MGs typically involves an in vitro or cell‐based binding assay.^[^ ^7^ ^]^ A number of in vitro methodologies have been exploited including fluorescence polarization,^[^ ^16^ ^]^ TR‐FRET,^[^ ^17^ ^]^ alpha‐screens,^[^ ^18^ ^]^ DNA‐encoded libraries^[^ ^19^ ^]^ and disulfide tethering.^[^ ^20^ ^]^ In cells, nano‐BRET,^[^ ^21^ ^]^ co‐immunoprecipitation,^[^ ^14^ ^]^ and proximity ligation methods^[^ ^22^ ^]^ have also been used. However, generation of structural data that can drive rational design approaches is largely reliant on protein X‐ray crystallography. Although hugely powerful, this is limited to systems amenable to crystallization, and only reports on a structural “snapshot”, not the dynamic protein landscape. Therefore **,** there is a need for viable methods not only to discover molecular glues, but also facilitate the rapid generation of structure‐activity relationships that can guide molecular glue optimization once an initial hit has been found.

To fully characterize molecular glues, it is crucial to utilize techniques that monitor the formation of molecular glue‐induced ternary complexes. Native mass spectrometry (native MS) is capable of distinguishing apo proteins from binary PPIs and monitor the formation of molecular glue‐stabilized ternary complexes.^[^ ^23^, ^24^, ^25^, ^26^, ^27^ ^]^ Previous work in our group has also highlighted the power of native MS in monitoring the time‐dependent formation of covalent molecular glue induced ternary complexes and uncovered the mechanism of action of a covalent molecular glue using further tandem MS experiments.^[^ ^28^ ^]^ Furthermore, by combining native MS with ion mobility spectrometry and specialized complex‐down MS approaches, additional information on ternary complexes can be revealed.^[^ ^29^ ^]^ Whilst native MS can uncover information regarding the stoichiometry, efficiency, specificity and mechanism of action of molecular glues, this method struggles to reveal the precise binding site of the molecular glue within the complex, or provide site specific information on how protein conformational dynamics changes upon molecular glue binding.

Hydrogen‐deuterium exchange mass spectrometry (HDX‐MS) is a powerful tool to investigate protein folding, protein dynamics and PPIs in solution.^[^ ^30^, ^31^ ^]^ The method utilizes time‐dependent, differential exchange of backbone amide hydrogens with deuterium in solution. The high resolving capability of mass spectrometers enables the detection of mass increases upon exchange. Backbone amide hydrogens involved in stabilizing hydrogen bonds exchange at a slower rate in comparison to solvent exposed backbone amide hydrogens or those involved in weak hydrogen bonds. The solvent accessibility decrease upon PPI formation, or induction/stabilization of PPIs, can be measured and, following digestion of the deuterated protein, the location of protein interfaces and ligand binding sites can be simultaneously determined.

**Figure 1:**
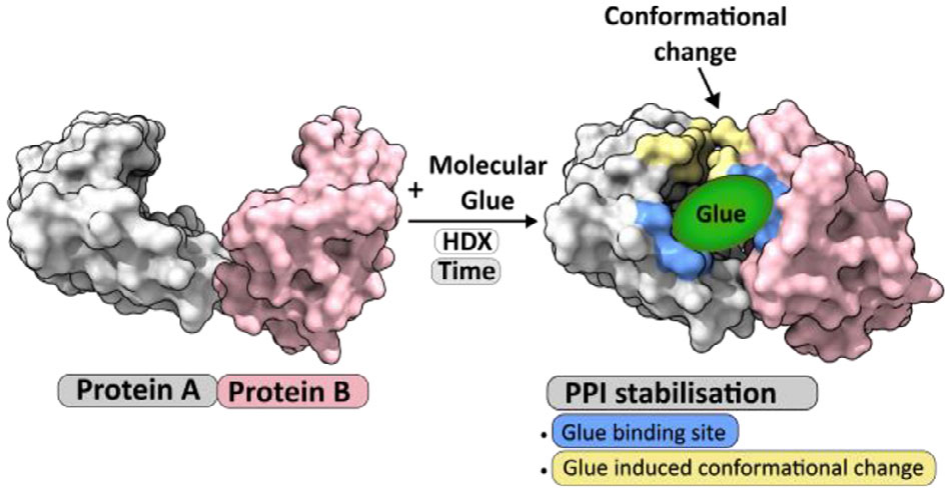
HDX‐MS is a valuable tool to characterize molecular glue‐induced stabilization of protein‐protein interactions. HDX‐MS can simultaneously provide information on the PPI interface location, the molecular glue binding site (highlighted in blue), protein dynamics, and any conformational changes induced by molecular glue binding (highlighted in yellow).

**Figure 2:**
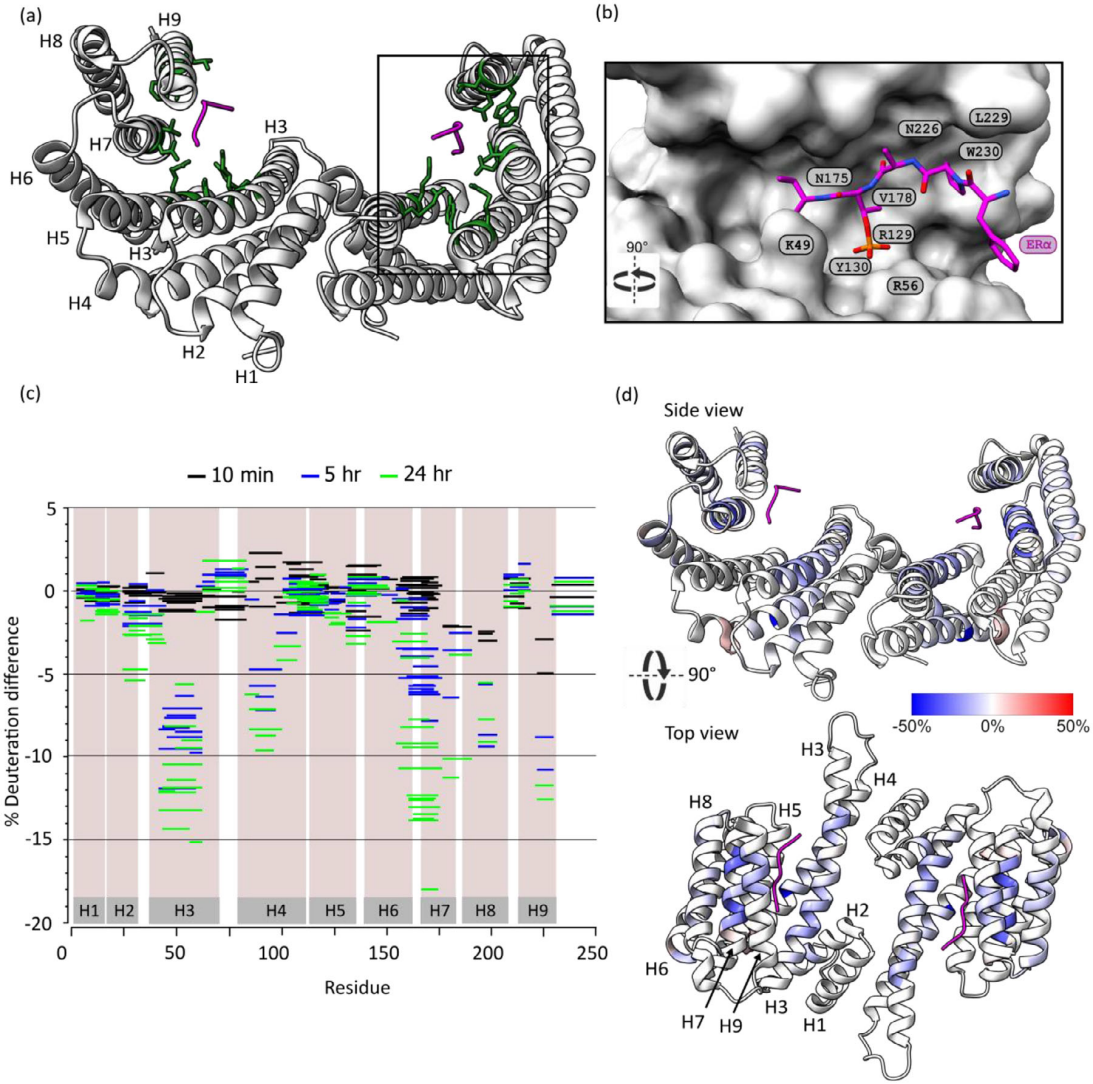
Protection from deuterium labelling occurs on 14‐3‐3σ upon ERα binding to 14‐3‐3σ. a) 14‐3‐3 is a dimeric protein with each monomer consisting of 9 α‐helices (H1‐H9). Peptide binding (shown in pink) occurs via polar and hydrophobic residues making up an amphipathic binding groove. Residues on 14‐3‐3 involved in binding are highlighted in green and consist of K49, R56, R129, Y130, N175, N226, W230, L299, and V178 b) Zoom in on the binding pocket with key residues highlighted. Binding of 14‐3‐3 with ‘mode 3′ binding partners (shown in pink) leaves a pocket available for FC‐A binding. c) Woods plot for visual comparison of the differences in deuterium incorporation within 14‐3‐3σ at 10 min (black), 5 h (blue), and 24 h (green) with and without ERα bound. d) HDX difference profile of 14‐3‐3σ bound to ERα minus 14‐3‐3σ alone after 24 h displayed on the 14‐3‐3σ crystal structure, PDB: 4JDD. Blue indicates protection from deuterium labeling.

HDX‐MS has been used to uncover step‐wise binding events^[^ ^32^ ^]^ and elucidate the binding affinity of protein‐drug interactions.^[^ ^33^ ^]^ Specific to bifunctional PROTACs and molecular glue degraders, HDX‐MS has been used to study induced ternary complex formation and to identify protein‐protein interfaces, or lack thereof, upon PROTAC/molecular glue binding.^[^ ^34^, ^35^, ^36^, ^37^ ^]^ Here, we showcase the utility of HDX‐MS for the analysis of molecular glues where it has the potential to identify interfaces and MG binding sites, and determine conformational changes upon molecular glue binding that contribute to the highly stabilized ternary complex (**Figure** **1**). We chose to focus on the molecular glue, Fusiccocin‐A (FC‐A) (Figure S2, Supporting Information); a fungal metabolite that enhances a subset of PPIs involving 14‐3‐3 proteins. 14‐3‐3s are dimeric eukaryotic hub proteins consisting of seven structurally homologous isoforms (β, γ, ε, η, σ, τ and ζ) that interact with over 1000 binding partners, typically in a phosphorylation‐dependent manner.^[^ ^38^, ^39^ ^]^ Each 14‐3‐3 monomer is made up of nine α‐helices, a highly disordered C‐terminus and a conserved amphipathic binding pocket, that accomodates the phosphorylated region of 14‐3‐3′s binding partner (**Figure** **2**a,b). The therapeutic benefit of targetting 14‐3‐3 PPIs with molecular glues has been well elucidated.^[^ ^40^, ^41^, ^42^ ^]^ Where a suitable binding pocket is present at the protein interface of the binary complex (Figure S3, Supporting Information),^[^ ^40^, ^43^, ^44^ ^]^ FC‐A binds and glues the PPI together. Structural characterization of the ternary complexes formed has been exclusively conducted via protein X‐ray crystallography of static structures. Therefore, we anticipated that HDX‐MS would provide new insight into the dynamic effects of FC‐A on 14‐3‐3 PPI systems.

## 2 Results and Discussion

First, we used HDX‐MS to characterize PPIs involving 14‐3‐3σ in the absence of molecular glue. Using peptide mimetics of the protein partners, we focussed on the 14‐3‐3σ interactions with estrogen receptor α (ERα_581‐595_pT594) and leucine‐rich repeat kinase 2 (LRRK2_928‐942_pS935) (termed ERα and LRRK2 hereafter). ERα is an important driver for breast cancer progression. Phosphorylation of ERα T594 creates a characteristic ‘mode 3’ binding motif for 14‐3‐3 that is amenable to FC‐A stabilization.^[^ ^45^, ^46^ ^]^ This 14‐3‐3 binding interaction prohibits ERα’s dimerization and subsequent transcriptional activity, thus impeding cancer cell growth.^[^ ^47^ ^]^ 14‐3‐3/LRRK2 PPI is implicated in Parkinson's disease where the binding of LRRK2 to 14‐3‐3 is impaired. LRRK2 interacts with 14‐3‐3 in a complex multivalent fashion.^[^ ^48^ ^]^ Ser935 phosphorylation generates an important interaction motif that is not amenable to FC‐A stabilization. It thus serves as a useful control (Figure S3, Supporting Information).

14‐3‐3σ was incubated in the presence and absence of ERα/LRRK2 for 10 min, 5 h, and 24 h. The reaction was quenched followed by online digestion of 14‐3‐3σ into peptides with pepsin/nepenthesin‐2 (Figure S4, Supporting Information). The peptides were then separated by liquid chromatography and the percentage deuterium incorporation was calculated at every time‐point for each peptide determined from the mass spectra. Deuterium exchange was observed in peptide regions that corresponded to known structural features of 14‐3‐3 (Figure S5, Supporting Information), with slow exchange rates reflecting the rigidity of the 14‐3‐3 protein structure. As expected, upon ERα binding we found regions of 14‐3‐3 that showed protection from deuterium exchange (Figure 2c). In particular, peptides located in helices H3, H7 and H9 showed significant levels of protection after 24 h (Figures 2c,d; S6, Supporting Information). These regions correlate with the 14‐3‐3 binding groove. These data are consistent with previous HDX‐MS studies investigating the interaction between 14‐3‐3ζ and a longer protein construct of the ERα ligand binding domain,^[^ ^46^ ^]^ 14‐3‐3γ and procaspase‐2,^[^ ^49^ ^]^ and the interaction between a yeast 14‐3‐3 isoform (Bmh1) and a neutral trehalase (Nth1).^[^ ^50^ ^]^ This confirms that the short, phosphorylated mimetic used here, makes similar primary contacts within the 14‐3‐3σ binding groove. Moreover, identical areas of protection were observed upon LRRK2 binding (Figure S7, Supporting Information), albeit the level of protection decreased relative to ERα, consistent with LRRK2s lower binding affinity for 14‐3‐3.^[^ ^48^ ^]^ Intriguingly, with ERα and LRRK2 binding, protection was also observed at H8, located distally from the binding groove (Figures 2d; S6 and S7, Supporting Information). This protection was also observed in previous studies where it was proposed that the protection could be a result of direct interactions of the ERα LBD/procaspase‐2 with the exterior of 14‐3‐3.^[^ ^46^, ^49^ ^]^ Our results, however, indicate that protection around H8 is more likely a result of allosterically induced changes in the 14‐3‐3 protein structure because the peptide mimetic bound to the 14‐3‐3 groove is too short to form direct contacts with H8. Indeed, a dynamic rotation movement of the three C‐ terminal α‐helices (H7, H8, H9) upon binding has been proposed as an integral feature of 14‐3‐3 molecular recognition and binding.^[^ ^51^, ^52^, ^53^, ^54^ ^]^

**Figure 3:**
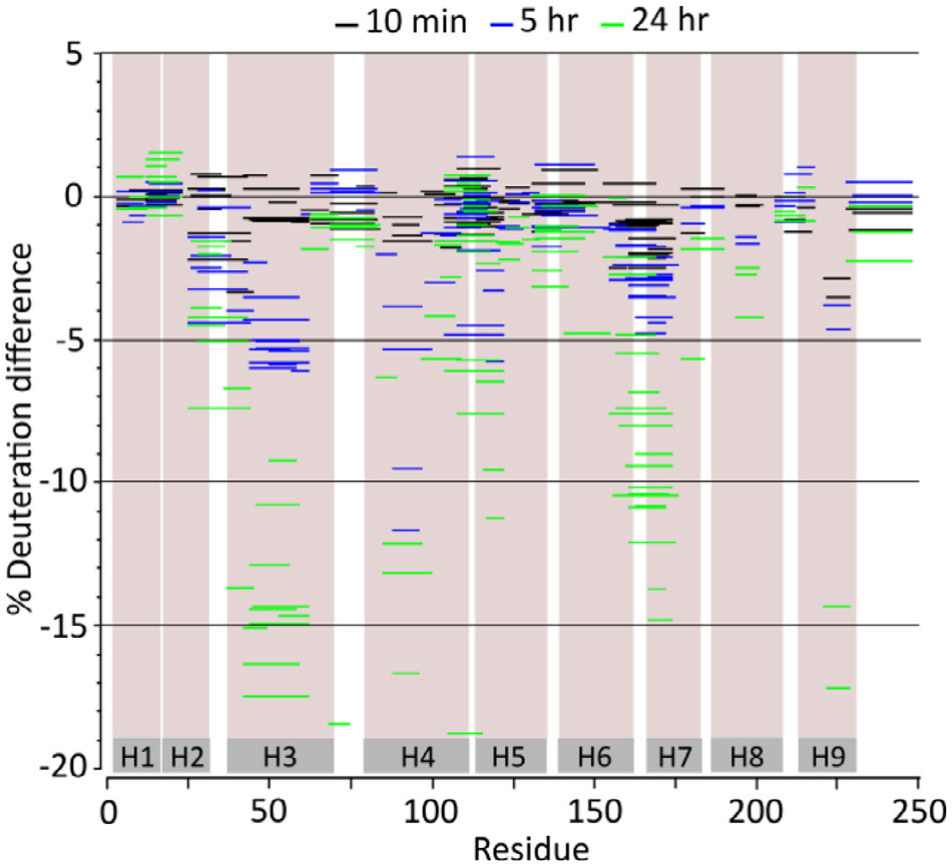
Regions on 14‐3‐3σ show additional protection from deuterium labelling upon molecular glue induced 14‐3‐3σ/ERα complex stabilization. a) Woods plot for visual comparison of the differences in deuterium incorporation within 14‐3‐3 at 10 min (black), 5 h (blue), and 24 h (green) for 14‐3‐3σ/ERα complex with and without the addition of molecular glue, FC‐A.

**Figure 4:**
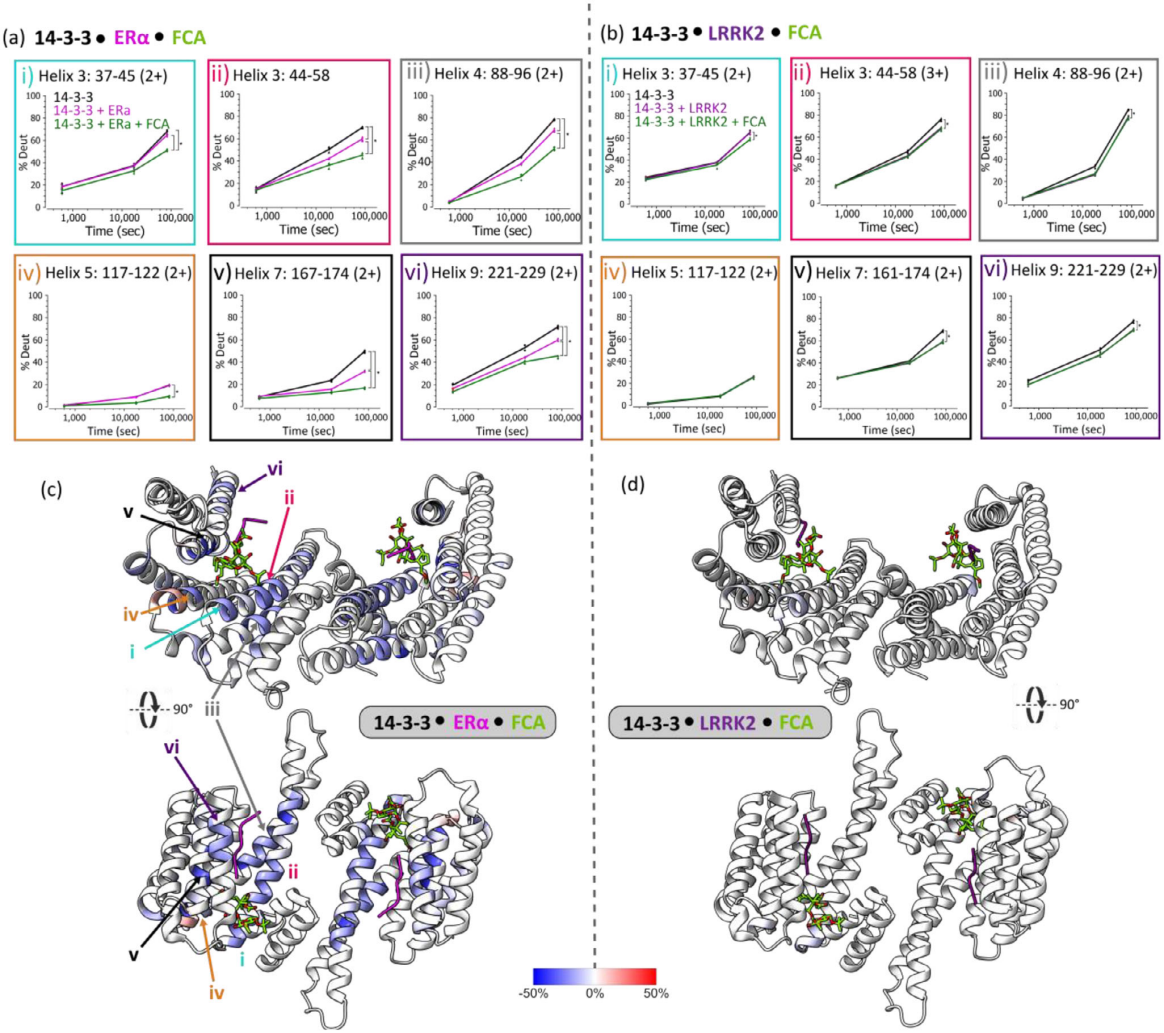
Protection from hydrogen‐deuterium exchange induced by molecular glue binding. a,b) Uptake plots (i‐vi) of six peptide regions of 14‐3‐3σ displaying % deuterium incorporation in the unbound (black), ERα/LRRK2 bound (pink/purple) and FC‐A stabilized (green) condition. HDX profiles involved with binding partners ERα and LRRK2 are displayed on the left and right, respectively. * represents significant differences between protein complex conditions at 24 h (*p* ≤ 0.05). c,d) HDX difference profile of 14‐3‐3/ERα (left) or 14‐3‐3/LRRK2 (right) with and without molecular glue, FC‐A at 24h mapped onto the 14‐3‐3σ structure (PDB: 4JDD (left), 5MY9 (right)). The location of the uptake plots of peptides i‐vi are labelled on the 14‐3‐3 structure.

Having established the key regions of 14‐3‐3 that elicit protection from deuterium exchange upon addition of its binding partner, we next investigated the influence of a molecular glue. We hypothesized that addition of FC‐A would elicit further protection from deuterium exchange as a result of increased ERα binding and complex stabilization. Indeed, when comparing deuterium labelling between the ERα bound complex and the molecular glue‐stabilized complex, additional protection was observed in several peptides across the 14‐3‐3σ sequence (**Figures** **3**; S8, Supporting Information). Mapping these peptides onto the crystal structure of 14‐3‐3 highlighted increased protection on H3, H7 and H9 consistent with an increased affinity of ERα for 14‐3‐3 upon addition of FC‐A (**Figures** **4**; S9, Supporting Information). In addition, we observed protection toward the N‐terminus of H3 and H5 between residues 37–45 and 117–122 (Figure 4a). This was not observed with ERα alone since that region of the 14‐3‐3 binding groove is unoccupied (Figure 2b). Therefore, additional protection in these regions was concluded to be a direct result of FC‐A binding. Previously, crystallography studies on the 14‐3‐3ζ /E6 oncoprotein interaction, showed the closure of H9 on 14‐3‐3ζ upon addition of FC‐A.^[^ ^55^ ^]^ Evidence of this H9 closure was not observed in our studies; it is an area of 14‐3‐3 that shows fast hydrogen‐deuterium exchange across all conditions (Figure S8, Supporting Information). This may reflect the different stabilization effect FC‐A has on its binding partners. Moreover, reorientation of the terminal helices of 14‐3‐3 has been observed with different structural orientations dependent on the size of its binding partners.^[^ ^51^, ^52^, ^53^, ^54^ ^]^ Another region of 14‐3‐3 that elicited additional protection from deuterium exchange upon FC‐A binding was between residues 85–100 on H4 and residues 26–36 on H2 (Figures 4; S9, Supporting Information). This region is located at the dimer interface of 14‐3‐3. Protection within this region suggests a reduction in dynamics and protein flexibility upon ERα binding (Figure S6, Supporting Information), that is then further amplified by the addition of FC‐A (Figure S9, Supporting Information). Indeed, the influence of protein partner binding on 14‐3‐3 dimerization has been investigated previously using FRET whereby titration of a tyrosine hydroxylase led to increased dimer stability.^[^ ^56^ ^]^

As previously mentioned, FC‐A elicits selectivity toward ‘mode 3′ interaction partners in which binding occurs with a C‐terminal XpS/TX_COOH_ motif of the client protein leaving a pocket adjacent to the client binding site for FC‐A (Figures 2; S3, Supporting Information). As such, it was expected that FC‐A would not glue the 14‐3‐3/LRRK2 complex because the partner protein chain extends through the FC‐A binding pocket in the binding groove^[^ ^48^ ^]^ (Figure S3, Supporting Information). To confirm this, we carried out HDX studies on the 14‐3‐3/LRRK2 complex in the presence and absence of FC‐A. No additional protection was observed in regions involved in the LRRK2 binding interface suggesting no stabilization effect (Figures 4; S11, Supporting Information). Very low levels of protection (∼8%) were afforded by residues 37 to 45 surrounding the FC‐A binding region (Figures 4b; S11, Supporting Information). This protection could be explained by a low affinity interaction between FC‐A and the 14‐3‐3/LRRK2 complex (Figure S14e, Supporting Information).

A key advantage of HDX‐MS is its ability to capture information on highly flexible regions of proteins that X‐ray crystallography cannot. In 14‐3‐3 proteins, the C‐terminus (residues 232–248) is highly disordered and as such, is not resolved in X‐ray crystal structures. Previous work using FRET has suggested a dynamic shift in the position of the C‐terminal region relative to 14‐3‐3′s binding groove upon protein partner binding, providing a specificity function toward 14‐3‐3 binding partners.^[^ ^57^, ^58^ ^]^ In our HDX‐MS study, we do not detect a change in C‐terminal deuterium incorporation in the presence and absence of protein partner or glue. Instead, we observe high deuterium incorporation at all time‐points and protein complex states (∼85%), consistent with the continued dynamics in the unbound and bound state. Moreover, this may reflect our HDX timescale from minutes to hours. Indeed, utilizing shorter timescales on the order of seconds or millisecond HDX^[^ ^59^, ^60^ ^]^ may resolve transient differences in hydrogen‐deuterium exchange that may be occurring at the 14‐3‐3′s C‐terminus.

## 3 Conclusion

Overall, we have highlighted the usefulness of applying HDX‐MS to characterize molecular glues. Using the model molecular glue system 14‐3‐3σ/ERα/FC‐A, we show that HDX‐MS can simultaneously determine the binding interface of molecular glues and showcase how molecular glues effectively stabilize PPIs. This information was captured on minute‐hour HDX‐MS timescales; however, it is important to optimize HDX‐MS timescales when applying this method to study alternate protein systems. Indeed, shorter timescales might be preferential to capture the most valuable information. We show that this HDX‐MS approach is highly feasible for the analysis of soluble protein oligomers (up to 60 kDa) whereby the Kd for the binary complex is in the low micromolar range (1.6 ± 0.3 µm for 14‐3‐3/ERα), and the molecular glue increases this affinity by >10‐fold (Kd = 0.1 ± 0.03 µm for 14‐3‐3/ERα in the presence of FC‐A.^[^ ^45^ ^]^ Although performed herein, the systematic analysis of the binary complex followed by the ternary complex system is not essential. Moreover, as long as the Kd for a given interaction in the presence of a molecular glue remains within the limits of HDX‐MS analysis (100–1000 µm,^[^ ^61^ ^]^), the differences in deuterium uptake between the ternary complex and an apo‐protein partner can provide valuable information on the ‘gluing’ mechanism. Due to HDX's ability to monitor any ternary complex system over a broad timescale, we envisage this approach will become a highly valuable tool in the characterization of molecular glues, guiding their optimization toward successful drug design.

## 4 Experimental Section

### Protein Expression and Purification

Recombinant His_6_‐tagged full‐length 14‐3‐3σ was expressed in BL21 (DE3) competent cells with a pET‐28a(+) plasmid. Expression was induced using IPTG. The cells were harvested and lysed by sonication. 14‐3‐3σ was purified from the cleared lysate using a combination of Ni^2+^‐affinity chromatography and size exclusion chromatography. The final 14‐3‐3σ protein purity was confirmed by a combination of SDS‐PAGE and native mass spectrometry analysis, and the final protein stored in 25 mM HEPES pH 7.5, 100 mm NaCl, and 10 mm MgCl_2_ at ‐80 °C until further use. For more details, see Supplementary Methods.

### Formation of Binary and Stabilized Complex for Native MS

LC‐MS grade dimethyl sulfoxide (DMSO) was purchased from Fisher Scientific. Lypholized ERα_581‐595_ 15mer peptide (Ac‐KYYITGEAEGFPA(pT)V‐COOH, Synpeptide Ltd) and lypholized LRRK2_928‐942_ 15mer peptide (Ac‐NLQRHSN(pS)LGPIFDH‐CONH_2_, Synpeptide Ltd) were diluted into 100 mM ammonium acetate to make peptide stocks of 1 mM and stored at ‐20 °C prior to use. Fusicoccin A (FC‐A) was provided as a gift from Yusuke Higuchi. It was obtained as a metabolite of wildtype Phomopsis amygdali and genetically modified Phomopsis amygdali Niigata‐2 as reported previously^[^ ^62^ ^]^ FC‐A stocks were stored in 100% DMSO at 10 m at ‐80 °C. 14‐3‐3 was buffer exchanged into 100 mM ammonium acetate (pH 6.8) using a 10 kDa molecular weight cut‐off Amicon Ultra centrifugal filter (Merck Millipore) and stored at ‐80 °C prior to use. To form the 14‐3‐3/ERα complex, 14‐3‐3 (5 µm) was incubated with ERα (1:1 ratio) in the presence and absence of FC‐A (50 µm) (1:1:10 ratio) for 10 min on ice or at 10 min, 5 h or 24 h at room temperature to mimic HDX conditions (Figure S16, Supporting Information). To form the 14‐3‐3/LRRK2 complex, 14‐3‐3 (5 µm) was incubated with LRRK2 (1:2 ratio) in the presence and absence of FC‐A (50 µm) (1:2:10 ratio). 14‐3‐3 binding ratios were optimized to enable single and double‐bound complex formation. 14‐3‐3 protein complexes were directly infused into the mass spectrometer. A final concentration of 0.5% DMSO was used for all experiments.

### Native Mass Spectrometry

All native MS experiments were performed on an Orbitrap Eclipse Tribrid mass spectrometer (Thermo Fisher Scientific) coupled to a nanoelectrospray source that used gold‐coated borosilicate glass capillaries, pulled in‐house. The instrument was calibrated with FlexMix (Thermo Fisher Scientific). Positive ionization mode was used throughout with the capillary voltage set to 1.2 kV. The source temperature was set at 275 °C, in‐source dissociation at 25, S‐lens RF at 100. High pressure mode was used and a mass range of 2000–8000 m/z used to monitor the binding equilibria. Mass spectra were acquired using a maximum ion injection time of 100 ms. The automatic gain control was set to 1 × 10^6^ and the ions detected in the Orbitrap with resolution set to 15,000. Data analysis was carried out using Xcalibur (v4.2). All proteins and protein complexes were identified based on their close matches to their theoretical mass (Table S1, Supporting Information). To quantify each complex, the relative abundance of the complexes observed was assumed to reflect their abundance in solution. The relative abundance of each complex was calculated as a percentage of the sum of all other complexes. All charge states observed for each complex were included in the calculations.

### Formation of Binary and Stabilized Complex for HDX‐MS

To form the 14‐3‐3/ERα complex, 14‐3‐3σ in 25 mm HEPES, 200 mm NaCl, and 10 mm MgCl_2_ pH 8.0 (100 µm) was incubated with ERα (100 µm) for 10 min. To form the stabilized complex, 14‐3‐3σ (100 µm) was incubated with ERα (100 µm) and FC‐A (1 mm) for 10 min on ice. To form the 14‐3‐3/LRRK2 complex, 14‐3‐3σ in 25 mm HEPES, 200 mM NaCl, and 10 mm MgCl_2_ pH 8.0 (100 µm) was incubated with LRRK2 (200 µm) for 10 min on ice. To form the stabilized complex, 14‐3‐3σ (100 µm) was incubated with LRRK2 (200 µm) and FC‐A (1 mm) for 10 min on ice. Control incubations with 14‐3‐3σ (100 µm) in isolation were also performed. A final concentration of 10% DMSO was used for all experiments.

### Deuterium Labeling of Protein Complex

Deuterium labelling was carried out by diluting 14‐3‐3, 14‐3‐3/ERα, 14‐3‐3/ERα/FC‐A, 14‐3‐3/LRRK2 and 14‐3‐3/LRRK2/FC‐A tenfold with 100 mM ammonium acetate 99.9% D_2_O pD 7.2 and incubating for 10 min, 5 h, and 24 h at room temperature. The reaction was quenched by removal of 50 µL protein complex at each time‐point and adding 30 µL 4 m urea, 200 mm potassium phosphate buffer, pH 2.4, followed by flash freezing in liquid nitrogen and stored at ‐80 °C. Non‐deuterated control samples were prepared by diluting 14‐3‐3, 14‐3‐3/ERα, 14‐3‐3/ERα/FC‐A, 14‐3‐3/LRRK2 and 14‐3‐3/LRRK2/FC‐A tenfold with 100 mM ammonium acetate followed by the addition of 30 µL quench buffer to 50 µL protein complex, followed by flash freezing and storage at ‐80 °C. The protein samples were thawed on ice by addition of 20 µL quench buffer followed by immediate LC‐MS analysis. 7.7 µg protein complex was injected onto a 50 µL sample loop. Each time point was analysed in triplicate by LC‐MS with the exception of the 10‐min timepoint for LRRK2, analysed in duplicate. Non‐deuterated control samples were analysed in triplicate by LC‐MS for ERα complexes and duplicate for LRRK2 complexes.

### Maximally Deuterated Sample Preparation

Maximally deuterated samples were prepared by buffer exchanging 14‐3‐3 into 100 mm ammonium acetate 99.9% D_2_O pD 7.2 at 20 °C using a 10 kDa molecular weight cut‐off Amicon Ultra centrifugal filter (Merck Millipore). Deuterated 14‐3‐3 was then diluted tenfold with 100 mm ammonium acetate 99.9% D_2_O pD 7.2 and formic acid (1% v/v) and incubated overnight in at 50 °C. The reaction was quenched with the addition of 30 µL 4 M urea, 200 mM potassium phosphate buffer, pH 2.4 to 50 µL MaxD_2_O reaction volume followed by flash freezing in liquid nitrogen and storage at ‐80 °C. Samples were thawed on ice by addition of 20 µL quench buffer followed by immediate LC‐MS analysis. 7.7 µg 14‐3‐3 was injected onto a 50 µL sample loop. Maximally deuterated samples were analyzed in triplicate.

### Liquid Chromatography

Samples were injected onto a vanquish UHPLC coupled to an Orbitrap Eclipse Tribrid mass spectrometer (Thermo Fisher Scientific) with a heated electrospray ionization source. A home‐built device was used for sample injection and column loading. Samples were first injected onto a dual nepenthesin‐2/pepsin POROS column (2.1 mm × 20 mm, 7 °C, Affipro) at a flow rate of 0.1 mL min^−1^ (MX‐Class Auxiliary Pump ThermoFisher) (ice‐cold 0.1% FA in water mobile phase). A dual protease column was chosen over a standard pepsin column to enhance protein digestion efficiency (from ≤ 88 to 96%) and expand on the diversity of peptides detected. Peptides following digestion were collected on a C18 trap (ACQUITY UPLC BEH 1.7 µm, 2.1 mm x 5 mm, Waters) and desalted for 3.5 min using a mobile phase consisting of ice‐cold 0.1% formic acid in water. Peptides were then eluted and separated with a C18 analytical column (Hypersil GOLD Vanquish, 50 mm x 2.1 mm, 1.9 µm, ThermoFisher) at a flow rate of 350 µL min^−1^ using mobile phase A (H_2_O and 0.1% formic acid) and mobile phase B (100% acetonitrile and 0.1% formic acid) and a gradient from 4% to 12% B over 0.3 min and from 12% to 40% B over the following 5.2 min. Eluted peptides were directly infused into the mass spectrometer. Mobile phases were kept on ice. The trap and analytical column were kept in an ice‐bath. Two blank (water and 0.1% formic acid) injections in‐between protein injections ensured the absence of sample carryover.

### Mass Spectrometry

Positive ionization mode was used throughout, with the capillary voltage set to 3.5 kV. The source temperature was set at 250 °C, vaporizer temperature at 50 °C and S‐lens RF at 60. For deuterated samples, full scan MS1 spectra were acquired in the Orbitrap mass analyzer using a resolution of 60,000, a mass range between 255 and 2,000 m/z. The maximum injection time and automatic gain control were set to auto. For control non‐deuterated samples, precursor ions were selected based on the Top 10 abundant ions and isolated in the quadrupole with a 2 m/z window. Higher‐energy collisional dissociation (HCD) with a normalized HCD energy of 30 was performed. Fragment ions were detected in the Orbitrap using a resolution setting of 30,000. For MS/MS experiments, the dynamic exclusion was employed for 4 s on a single‐charge state per precursor, and only charge states from 2+ to 10+ were selected for MS/MS.

### Data and Statistical Analysis

Non‐deuterated RAW files were processed using Proteome Discoverer v2.5. (Thermo Fisher Scientific). For all searches, enzyme cleavage was set to semi, and the maximum number of missed cleavages set to 12 with a minimum peptide length of 6. A precursor mass tolerance of 10 ppm was used, with a fragment mass tolerance of 0.02 Da. The peptide validator node was set to filter for a false discovery rate of 0.01. Sequence files that were included in the search comprised of 14‐3‐3σ, pepsin and nepenthesin‐2. The peptide identification list was established with apo 14‐3‐3, reporting all peptides identified across three replicates. Note: that no decrease in sequence coverage was observed upon binary/ternary complex formation. The peptide list and corresponding retention times were exported and subsequent data analysis carried out in HDExaminer (v3.4.2, Sierra Analytics, Modesto, CA). Peptides were filtered based on their matches to the theoretical isotopic distribution, consistency with overlapping peptides and charge states and spectrum crowding. Peptides with >20 residues were excluded from analysis. Deuterium values were calculated using the charge state with the best spectral match to the theoretical isotopic distribution. To assess the statistical significance of the % deuterium difference between protein complex states (n = 3), an unpaired student's t‐test was carried out. An uptake difference of 0.41 Da, 0.33 Da and 0.44 Da was used for the comparison between apo 14‐3‐3 and 14‐3‐3/ERα, 14‐3‐3/ERα and 14‐3‐3/ERα/FC‐A, and apo 14‐3‐3 and 14‐3‐3/ERα/FC‐A, respectively to determine a significant fold‐change (Table S2, Supporting Information). For LRRK2, an uptake difference of 0.65 Da, 0.68 Da and 0.66 Da was used for the comparison between apo 14‐3‐3 and 14‐3‐3/LRRK2, 14‐3‐3/LRRK2 and 14‐3‐3/LRRK2/FC‐A, and apo 14‐3‐3 and 14‐3‐3/LRRK2/FC‐A, respectively (Table S2, Supporting Information). The non‐significant differences (both p value ≥ 0.05 and fold change less than the determined value) in the difference heat maps are labelled as 0. Chimera (v1.9) was used to label the difference heat maps onto 14‐3‐3 crystal structures (pdb 4JDD and 5MY99).

Deuteration levels for each peptide at each time‐point (% D) were determined using (Equation 1).

(1) $$\%D=\frac{\left(m-m_{0}\right)}{\left(m_{100}-m_{0}\right)}\times 100$$

where m is the experimental peptide centroid mass, m_0_ is the non‐deuterated peptide centroid mass and m_100_ is the maximally labelled peptide centroid mass.

Due to the home‐built set up of the device it was important to determine the low levels of back‐exchange. We determined a value of 29.6% back‐exchange (22.6%‐39.3%) using a sample of 20 peptides across the entire 14‐3‐3 sequence. Back exchange for each peptide was calculated using HDExaminer software with (Equation 2).

(2) $$\mathrm{Back}\mathrm{exchange}=\left(1-\frac{m_{100}-m_{0}}{N\times D_{frac}}\right)\times 100$$

where m_100_ was the maximally labelled peptide centroid mass, m_0_ is the non‐deuterated peptide centroid mass, N is the theoretical number of exchangeable amides in the peptide (excluding proline), and D_frac_ is the fraction of D/H in the labelling buffer used. Back‐exchange was calculated from maximally deuterated 14‐3‐3 (>99%), as opposed to 90% deuterated 14‐3‐3 that was the maximum deuterium content feasible within experiments taking into account the tenfold dilution during sample preparation.

## Conflict of Interest

The authors declare no conflict of interest.

## Supporting information



Supporting Information

## Data Availability

The data that support the findings of this study are openly available in PRIDE at https://doi.org/**10.6019/PXD065689**, dataset identifier PXD065689.
