# Supplementary material for: Hydrogen‐Deuterium Exchange Mass Spectrometry for Molecular Glue Characterization
Source: Adv Sci (Weinh). 2025 Aug 4;12(41):e08543. doi: 10.1002/advs.202508543 (PMC12591169; doi:10.1002/advs.202508543)
Supplement: Supplementary file 1 — Supporting Information [file ADVS-12-e08543-s001.docx]

**Supporting Information**

Hydrogen-Deuterium Exchange Mass Spectrometry for Molecular Glue Characterization

Danielle F. Kay,^1^ Hadeeqa G. Raza,^1,2^ Richard G. Doveston,^2^ Aneika C. Leney*^1^

^1.^ School of Biosciences, University of Birmingham, Birmingham, UK

^2.^ Institute of Structural and Chemical Biology and School of Chemistry, University of Leicester, Leicester, UK

Table of Contents

Experimental Section

Protein expression and purification 4

Supplementary Figures

Figure S1: Sequence of 14-3-3σ protein . 5

Figure S2: Chemical structure of FC-A. 5

Figure S3: Location of FC-A binding site on 14-3-3•ERα protein complex. 6

Figure S4: Peptide coverage of 14-3-3σ protein during HDX-MS. 7

Figure S5: Mirror plot showing protection from deuterium labelling on 14-3-3σ 8

upon ERα binding.

Figure S6: HDX difference profile for 14-3-3σ with and without ERα bound. 9

Figure S7: HDX difference profile for 14-3-3σ with and without LRRK2 bound. 10

Figure S8: Mirror plot showing protection from deuterium labelling on 14-3-3σ 11

-ERα complex upon FC-A induced stabilization.

Figure S9: HDX difference profile on 14-3-3σ comparing the 14-3-3σ/ERα 12

bound complex vs 14-3-3σ/ERα/FC-A stabilized complex.

Figure S10: Deuterium uptake plots comparing apo 14-3-3σ, 14-3-3σ/ERα and 13-14

14-3-3σ/ERα/FC-A stabilized complex.

Figure S11: HDX difference profile on 14-3-3σ comparing the 14-3-3σ/LRRK2 15

bound complex vs 14-3-3σ/LRRK2/FC-A bound complex.

Figure S12: HDX difference profile on 14-3-3σ comparing apo 14-3-3σ and 16

14-3-3σ/ ERα/FC-A stabilized complex.

Figure S13: Native MS of apo 14-3-3σ, 14-3-3σ/ERα bound complex 17

and 14-3-3σ/ERα/FC-A stabilized complex.

Figure S14: Native MS of apo 14-3-3σ, 14-3-3σ/LRRK2 bound 18

complex and 14-3-3σ/LRRK2/FC-A bound complex.

Figure S15: Deuterium uptake plots comparing apo 14-3-3σ, 14-3-3σ/LRRK2 19-20

and 14-3-3σ/LRR2/FC-A bound complex.

Figure S16: Native MS of 14-3-3σ/ERα/FC-A stabilized complex 21

under conditions mimicking HDX-MS.

Supplementary Tables

Table S1: Theoretical and measured masses of protein complexes. 22

Table S2: HDX-MS sample information. 23

Table S3: Raw HDX-MS data for apo 14-3-3σ, 14-3-3σ/ERα and 24

14-3-3σ/ERα/FC-A

References 27

**Experimental Section**

**Protein expression and purification.**

Recombinant His_6_-tagged full-length 14-3-3σ was expressed in BL21 (DE3) competent cells with a pET-28a(+) plasmid. A single transformed colony was used to inoculate 20 mL terrific broth (containing 50 µg/mL kanamycin) which was grown overnight at 37 °C, 180 rpm. The starter culture was used to inoculate 1 L of terrific broth media (containing 50 µg/mL kanamycin) and supplemented with 5 mM MgCl_2_. The cells were grown at 37 °C, 180 rpm until the OD_600_ reached 0.6 – 0.8. Expression was induced by the addition of 0.4 mM IPTG. Incubation was continued overnight at 25 °C, 180 rpm.

Cells were harvested by centrifugation (5,000 rpm, 4 °C, 20 mins) and resuspended in buffer consisting of 50 mM HEPES pH 8.0, 300 mM NaCl, 12.5 mM Imidazole supplemented with DNAase and a Pierce^TM^ protease inhibitor tablet. The cells were lysed by sonication and the addition of lysozyme. The lysate was cleared by centrifugation (13,000 rpm, 4 °C, 50 mins). The clear lysate was loaded onto a Ni^2+^-affinity chromatography column equilibrated with 50 mM HEPES pH 8.0, 300 mM NaCl and 12.5 mM imidazole. The Ni^2+^-affinity chromatography column was washed with 50 mM HEPES pH 8.0, 300 mM NaCl and 25 mM imidazole. The protein was eluted with 50 mM HEPES pH 8.0, 300 mM NaCl and 250 mM imidazole. The protein was dialysed against buffer containing 25 mM HEPES pH 7.5, 100 mM NaCl and 10 mM MgCl_2_ and concentrated using a 10 kDa cut-off centrifugal filter unit (Merck Millipore). It was further purified by size-exclusion chromatography using a Superdex 75 30/100 HiLoad column (GE Healthcare). Protein-containing fractions were concentrated to 500 µM using a 10 kDa cut-off centrifugal filter unit (Merck Millipore).

**Supplementary Figures and Tables**


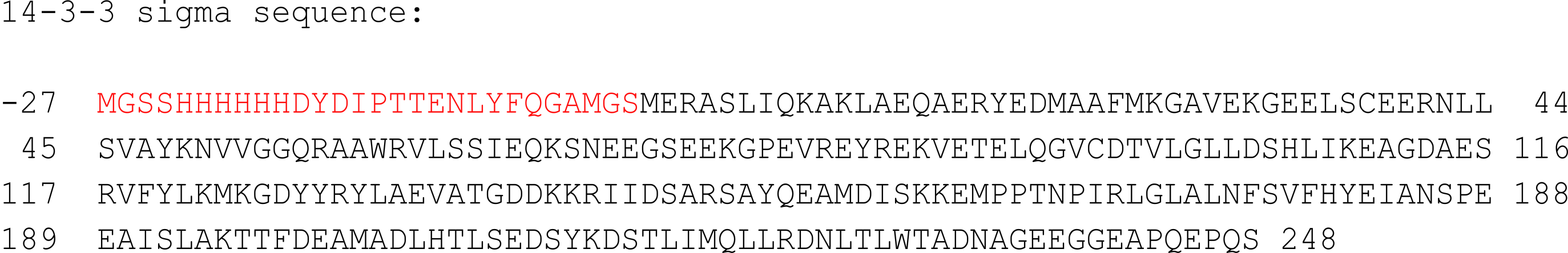


Fig. S1 Sequence of 14-3-3σ purified and expressed with a His-tag. The His-tag is highlighted in red. Peptides generated from online protein cleavage using a dual protease column corresponding to the His-tag were not included in downstream HDX-MS analysis.


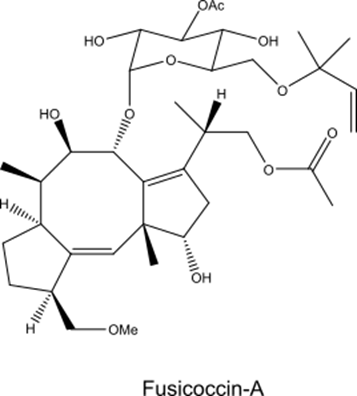


Fig. S2 Chemical structure of fusicoccin-A (FC-A).

**
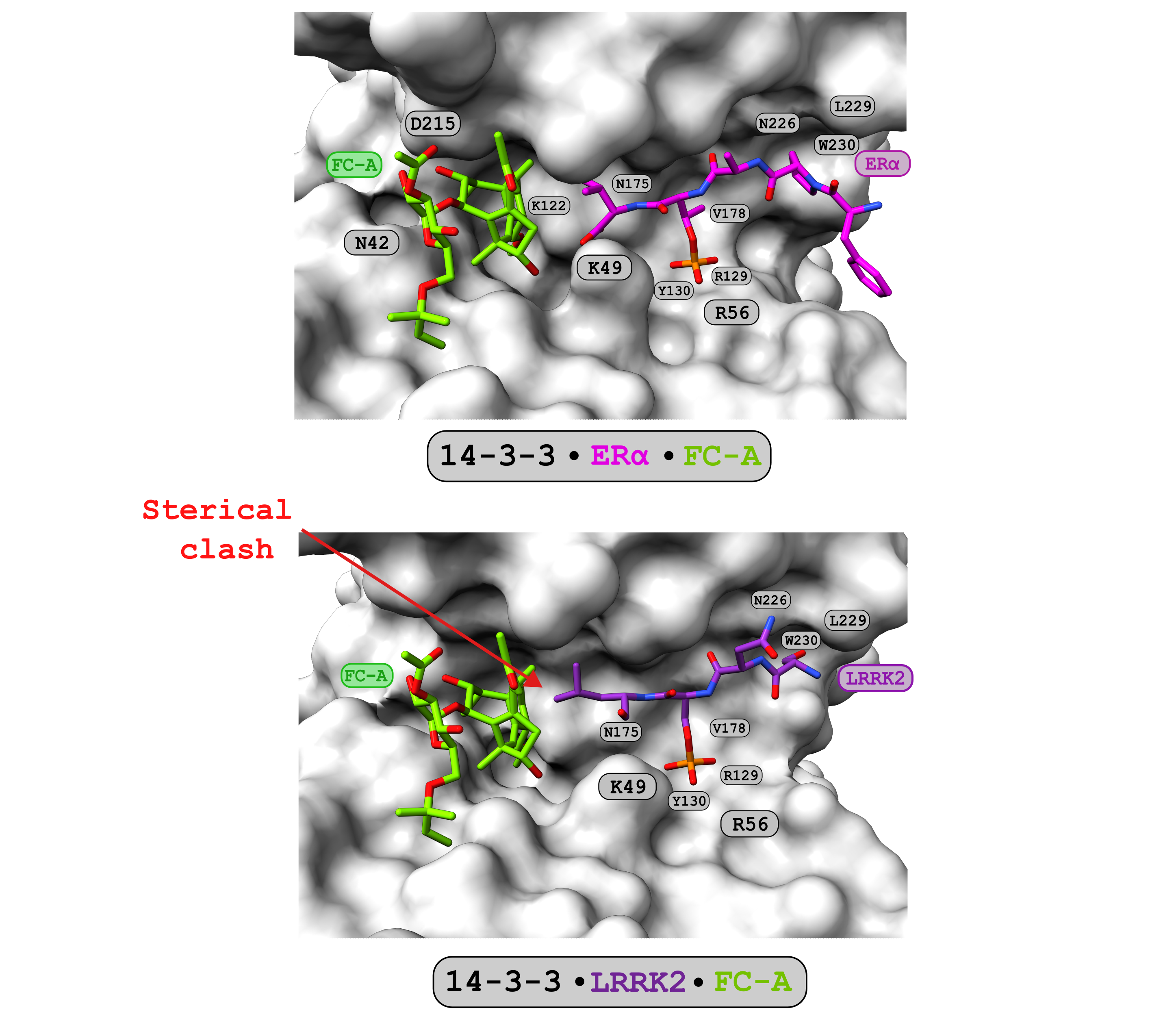
**

**Fig. S3** **FC-A binds and stabilizes the 14-3-3•ERα protein complex but does not stabilize the 14-3-3•LRRK2 complex because the protein partner occupies the FC-A binding site.** Top: Insert of the amphipathic binding groove of 14-3-3 showing binding of FC-A (green) to the 14-3-3•ERα complex (pink) (PDB 4JDD). 14-3-3σ residues involved in binding FC-A and ERα are labelled in grey. N42, D215 and K122 interact with FC-A. Bottom: Insert of the amphipathic binding groove of 14-3-3 accommodating LRRK2 (purple) which extends through the binding groove (electron density not seen) (PDB 5MY9). For illustrative purposes, FC-A (green) is positioned to highlight how FC-A would have to compete for binding with the continuing LRRK2 peptide sequence.

**
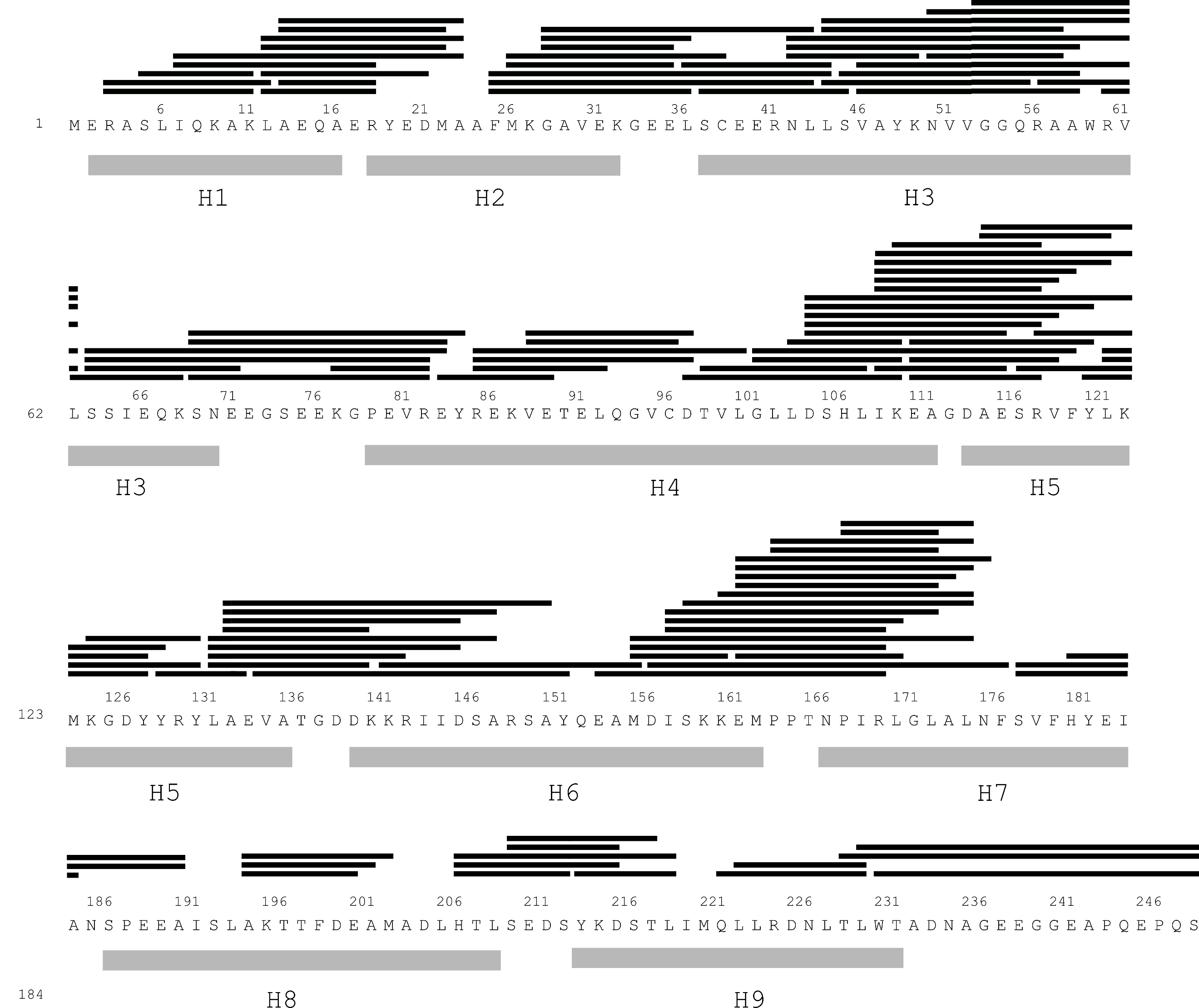
**

**Fig. S4. Peptide coverage of 14-3-3σ following online digestion with a dual pepsin/nepenthesin-2 column.** Peptides are illustrated by black lines above the 14-3-3 sequence and corresponding residue numbers. The nine α-helices of 14-3-3 are shown in grey boxes below the protein sequence and are labeled H1-H9. A total number of 124 peptides were analysed for deuterium uptake providing a sequence coverage of 96.4% with a redundancy of 5.4.


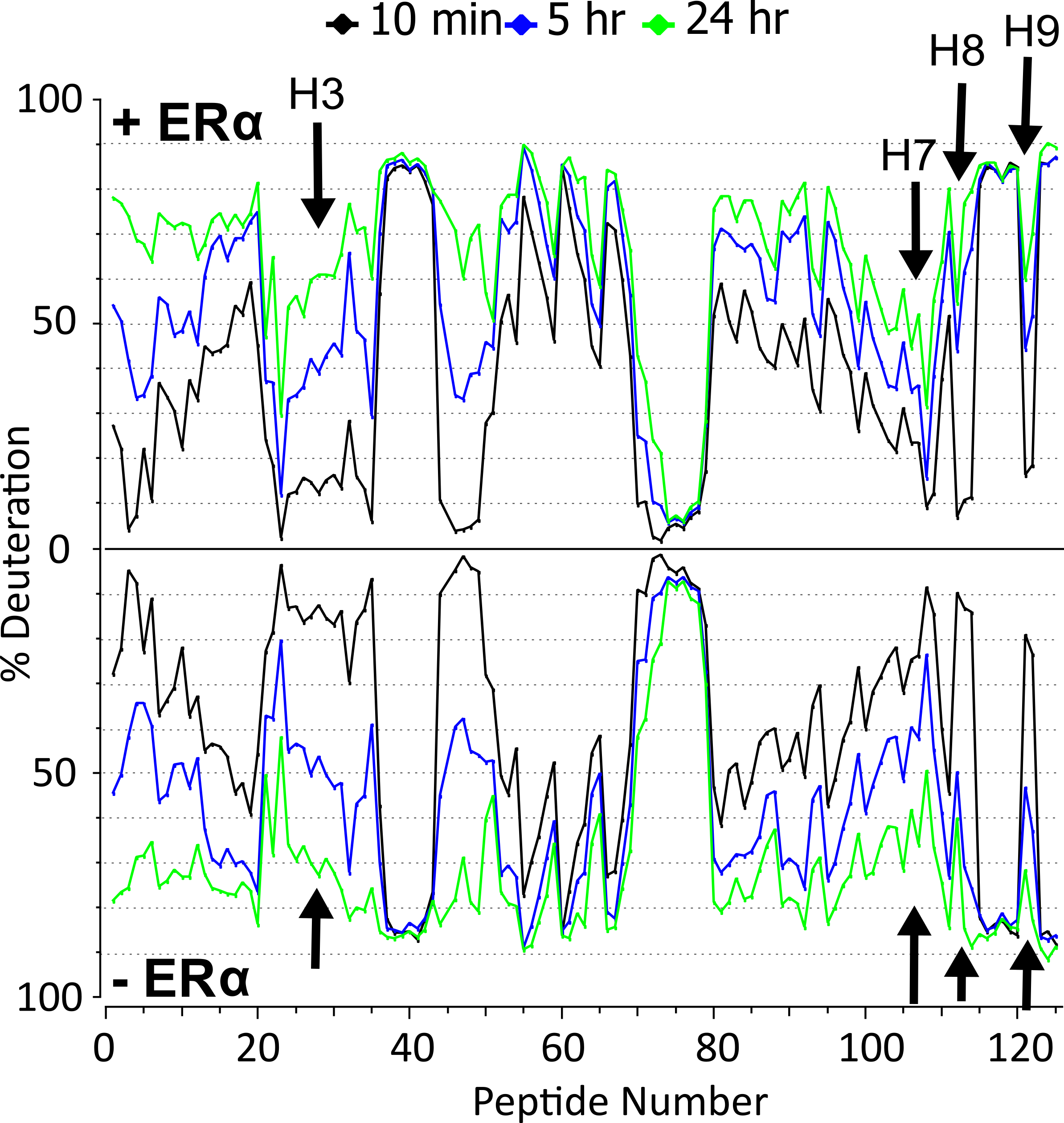


**Fig. S5. Protection from deuterium labelling occurs on 14-3-3σ upon ERα binding to 14-3-3σ.** Mirror plot for visual comparison of % deuterium incorporation within 14-3-3σ at 10 min (black), 5 h (blue) and 24 h (green) with (top) and without (bottom) ERα bound.

**
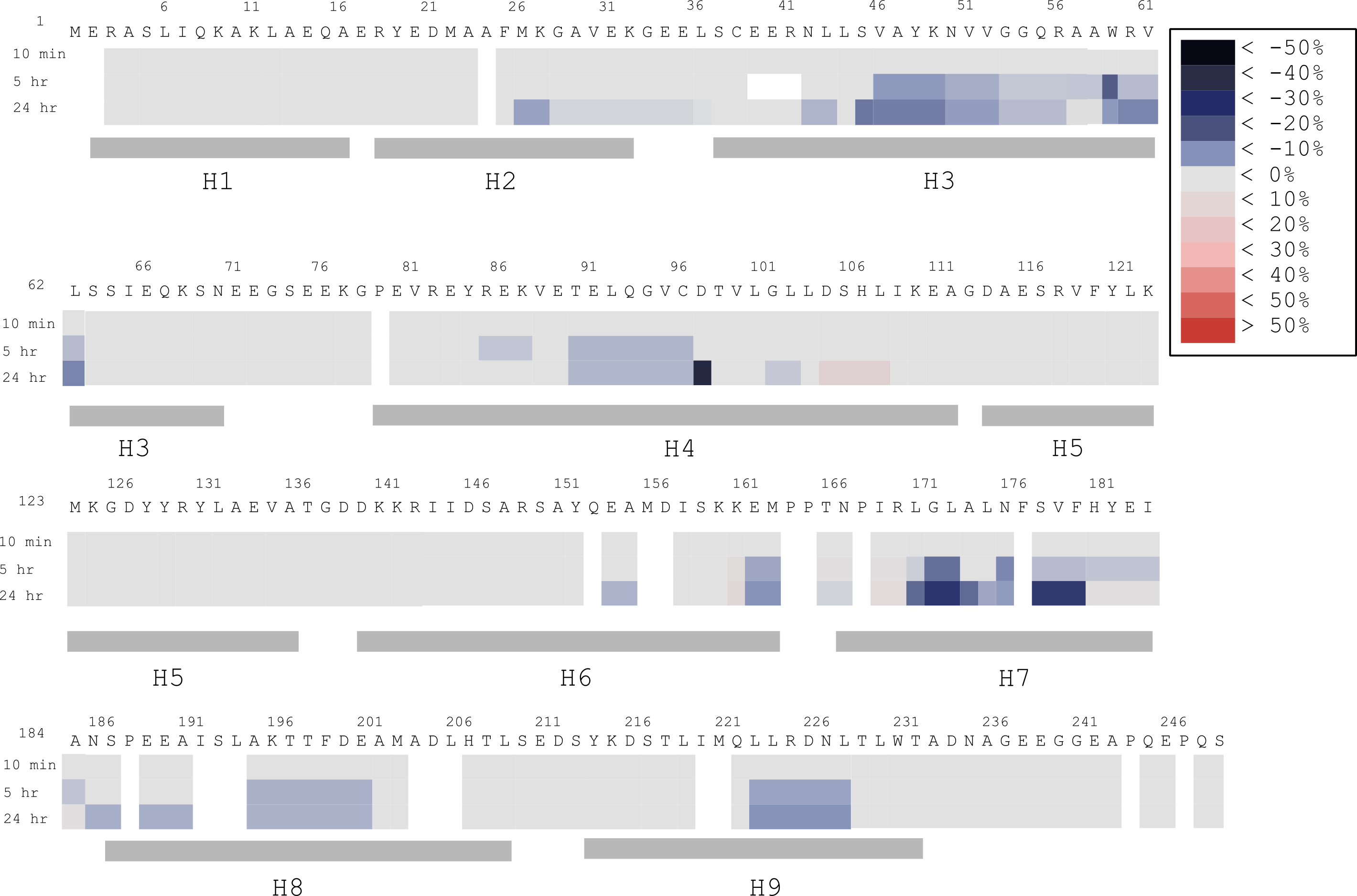
**

**Fig. S6. HDX difference profile for 14-3-3σ with and without ERα bound.** Blue represents regions protected from deuterium exchange whilst red represents deprotected regions. Each line represents a different time-point (10 minutes, 5 hours and 24 hours). α-helices are labelled (H1-9). Non-significant differences between the protein complex conditions (p ≥ 0.05) are labelled as 0.

**
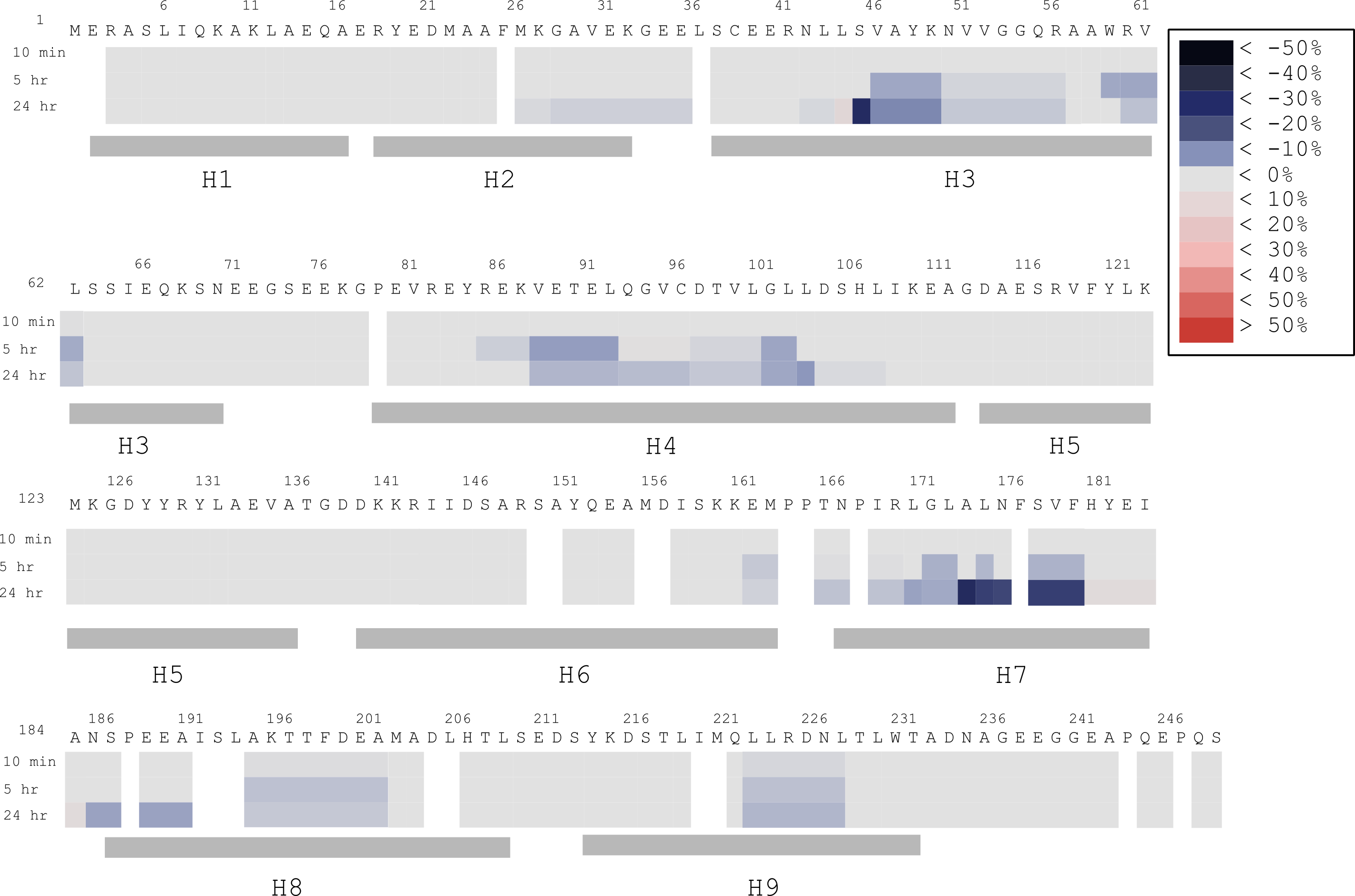
 Fig. S7. HDX difference profile for 14-3-3σ with and without LRRK2 bound.** Blue represents regions protected from deuterium exchange whilst red represents deprotected regions. Each line represents a different time-point (10 minutes, 5 hours and 24 hours). α-helices are labelled (H1-9). Non-significant differences between the protein complex conditions (p ≥ 0.05) are labelled as 0.


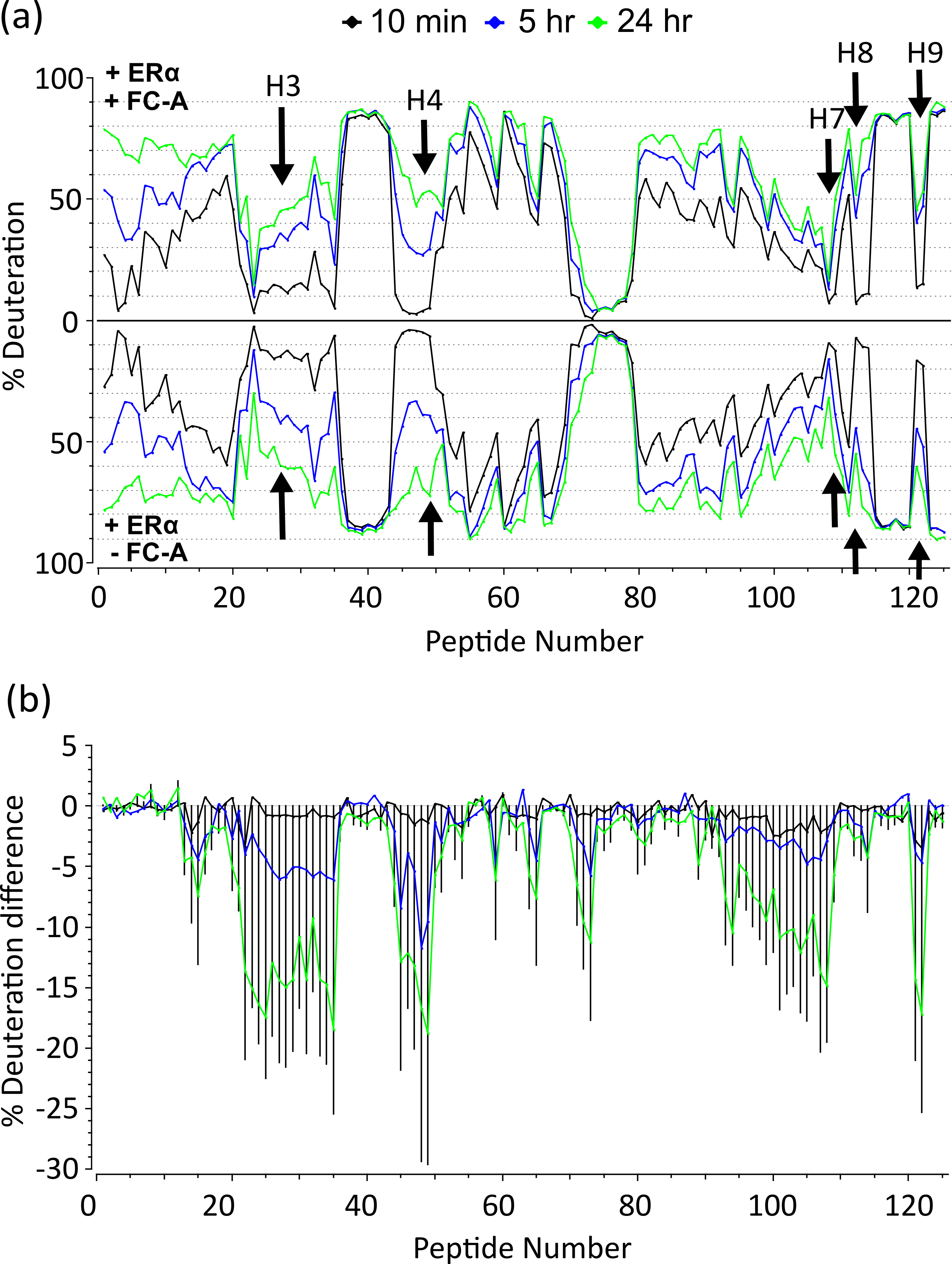
**Fig. S8.** **Regions on 14-3-3σ show additional protection from deuterium labelling upon molecular glue induced 14-3-3σ/ERα complex stabilization.** (a) Mirror plot for visual comparison of the differences in % deuterium incorporation within 14-3-3σ at 10 min (black), 5 h (blue) and 24 h (green) for 14-3-3σ/ERα complex with (top) and without (bottom) the addition of molecular glue, FC-A. (b) Difference plot calculated from data in (a) indicates that the main differences in HDX uptake are in peptides 23–35, 45-51, 71-73, 92-114 and 120-122. Black lines represent the total measured difference between the 14-3-3σ/ERα and 14-3-3σ/ERα/FC-A complex for each peptide over all time points.

**
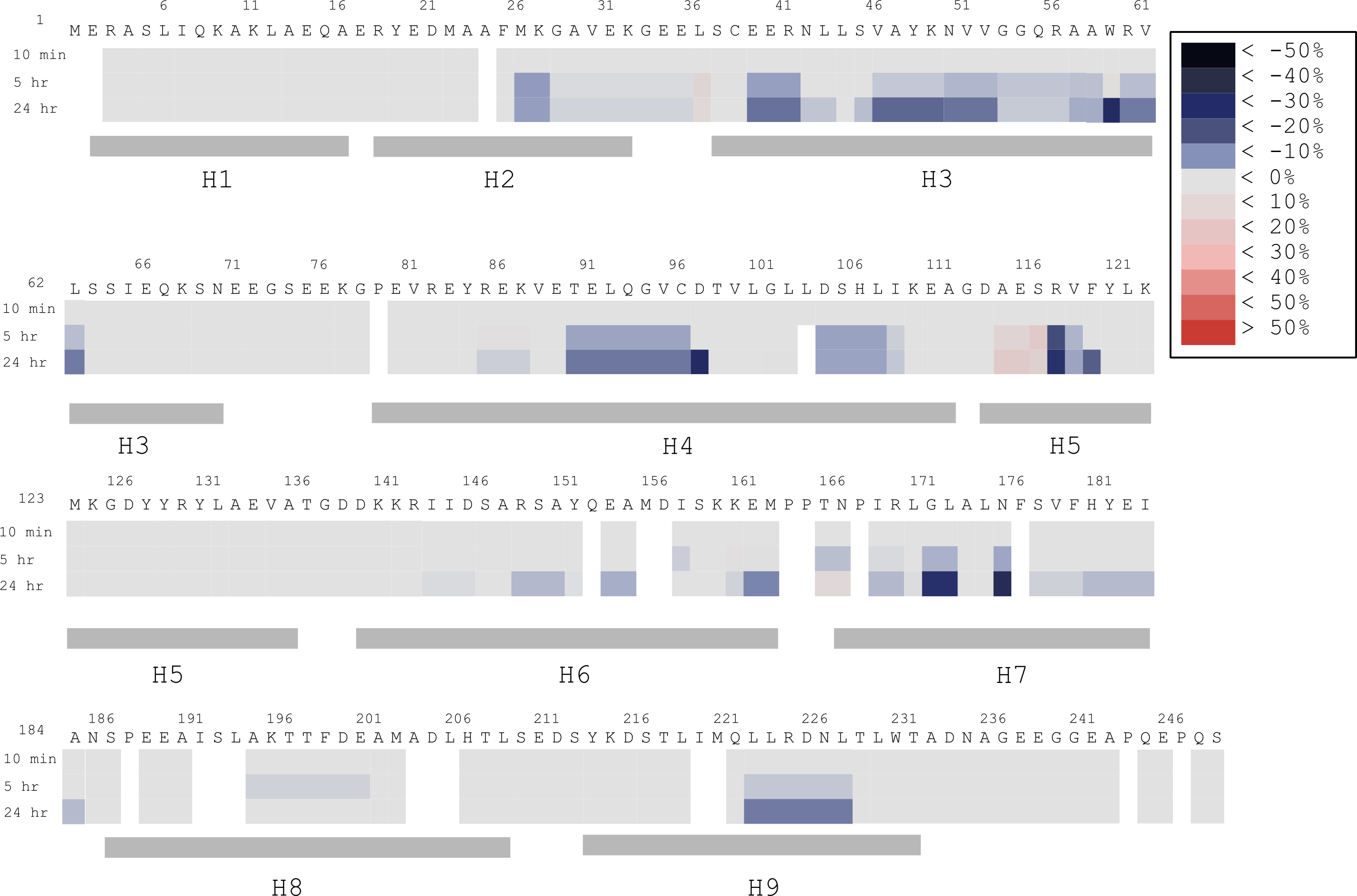
**

**Fig. S9.** **HDX difference profile on 14-3-3σ comparing the 14-3-3σ/ERα bound complex vs 14-3-3σ/ERα/FC-A stabilized complex.** Blue represents regions protected from deuterium exchange whilst red represents deprotected regions. Each line represents a different time-point (10 minutes, 5 hours and 24 hours). α-helices are labelled (H1-9). Non-significant differences between the protein complex conditions (p ≥ 0.05) are labelled as 0.


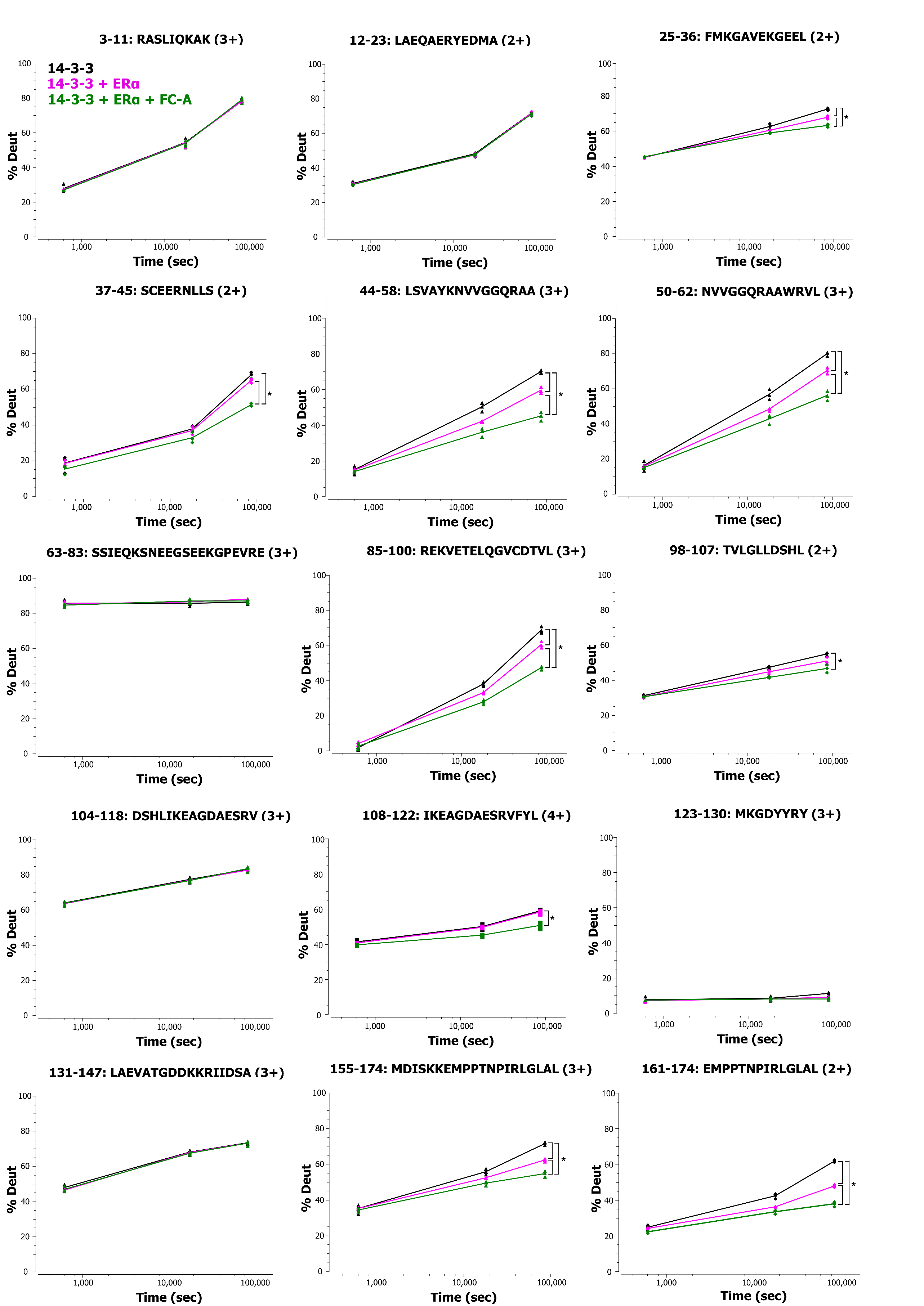


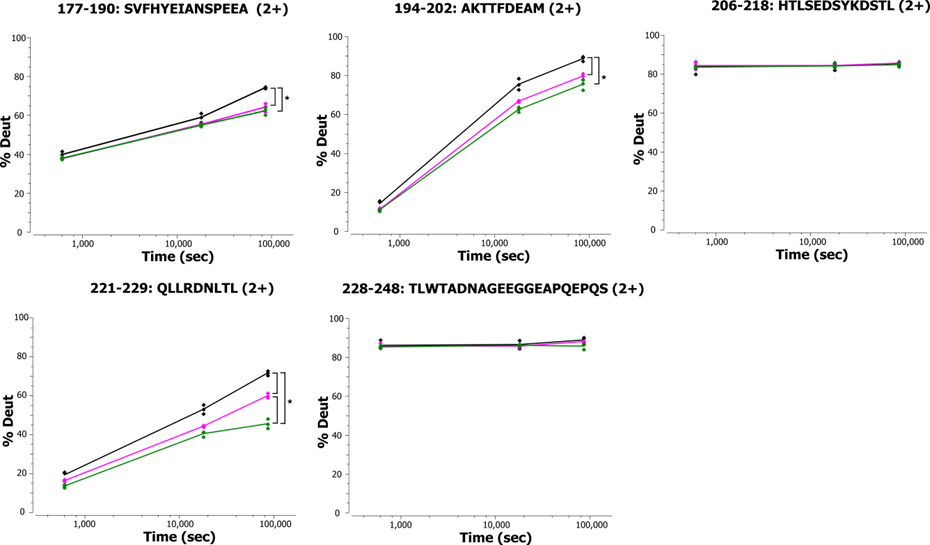


**Fig. S10.** Uptake plots of peptides covering the 14-3-3σ sequence displaying % deuterium (% Deut) incorporation at 10 minutes, 5 hours and 24 hours in the unbound (black), Erα bound (pink) and FCA stabilized (green) complex. 20 out of 124 total peptides were selected to show deuterium uptake over time. The 20 peptides cover the majority of the 14-3-3 sequence. Peptide sequences and their residue numbers are shown above the plots with the corresponding charge state. * represents significant differences between protein complex conditions at 24 hours (p ≤ 0.05).

**
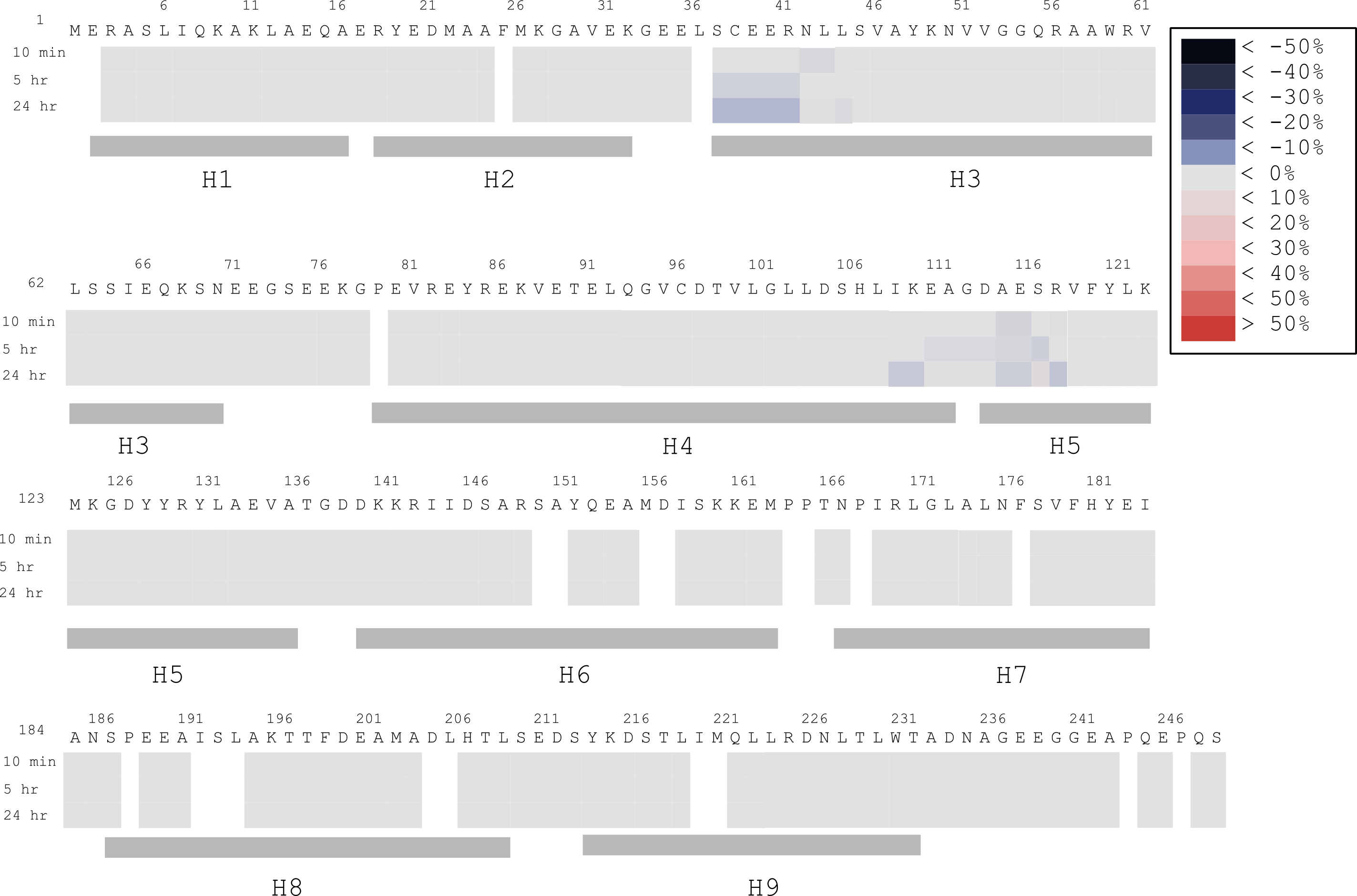
**

**Fig. S11. HDX difference profile on 14-3-3σ comparing the 14-3-3σ/LRRK2 bound complex vs 14-3-3σ/LRRK2/FC-A bound complex.** Blue represents regions protected from deuterium exchange whilst red represents deprotected regions. Each line represents a different time-point (10 minutes, 5 hours and 24 hours). α-helices are labelled (H1-9). Non-significant differences between the protein complex conditions (p ≥ 0.05) are labelled as 0.

**
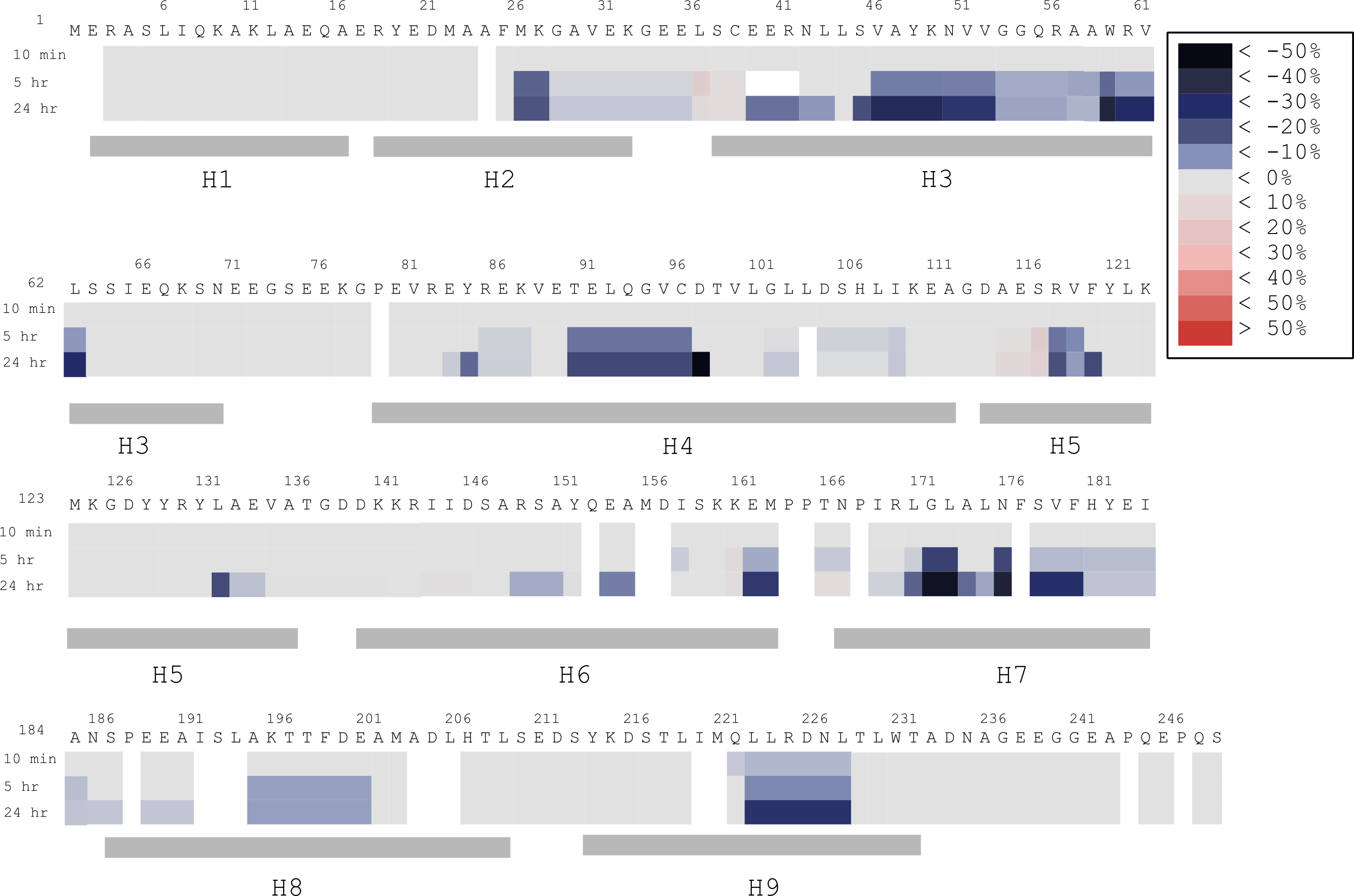
**

**Fig. S12. HDX difference profile on 14-3-3σ comparing unbound 14-3-3 vs 14-3-3σ/ERα/FC-A stabilized complex.** Blue represents regions protected from deuterium exchange whilst red represents deprotected regions. Each line represents a different time-point (10 minutes, 5 hours and 24 hours). Alpha helices are labelled (H1-9). Non-significant differences between the protein complex conditions (p ≥ 0.05) are labelled as 0.

**
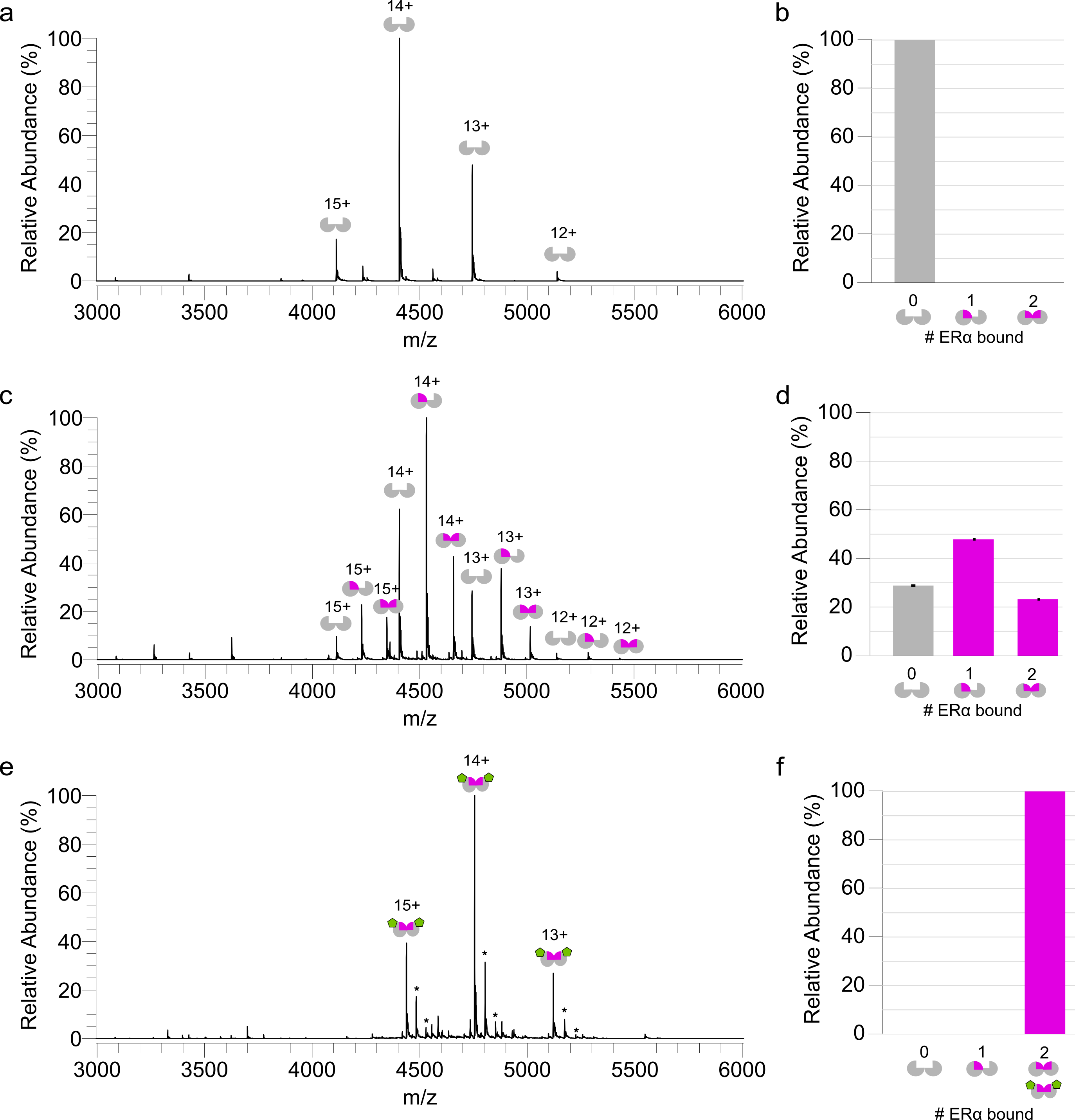
**

**Fig. S13. Native mass spectra showing that FC-A stabilizes the interaction between 14-3-3σ and ERα.** Native mass spectra of 14:3:3 alone (a), 14:3:3 with ERα in a 1:1 ratio (b), and 14:3:3 with ERα and FC-A in a 1:1:10 14-3-3:ERα:FC-A ratio (c). The fraction of bound complexes observed are shown in b, d, and f, respectively. In the absence of FC-A, singly and doubly bound ERα is observed in complex with 14-3-3. Upon addition of FC-A, the binding equilibrium is shifted to the fully stabilized ternary complex. * likely represents non-specific adducts of FC-A that form during the electrospray ionisation process due to the high concentrations of FC-A being used however, non-specific interactions in solution cannot be ruled out. The average relative abundance of each protein complex across 3 replicates is shown in the bar charts with the error reported as standard deviation.

**
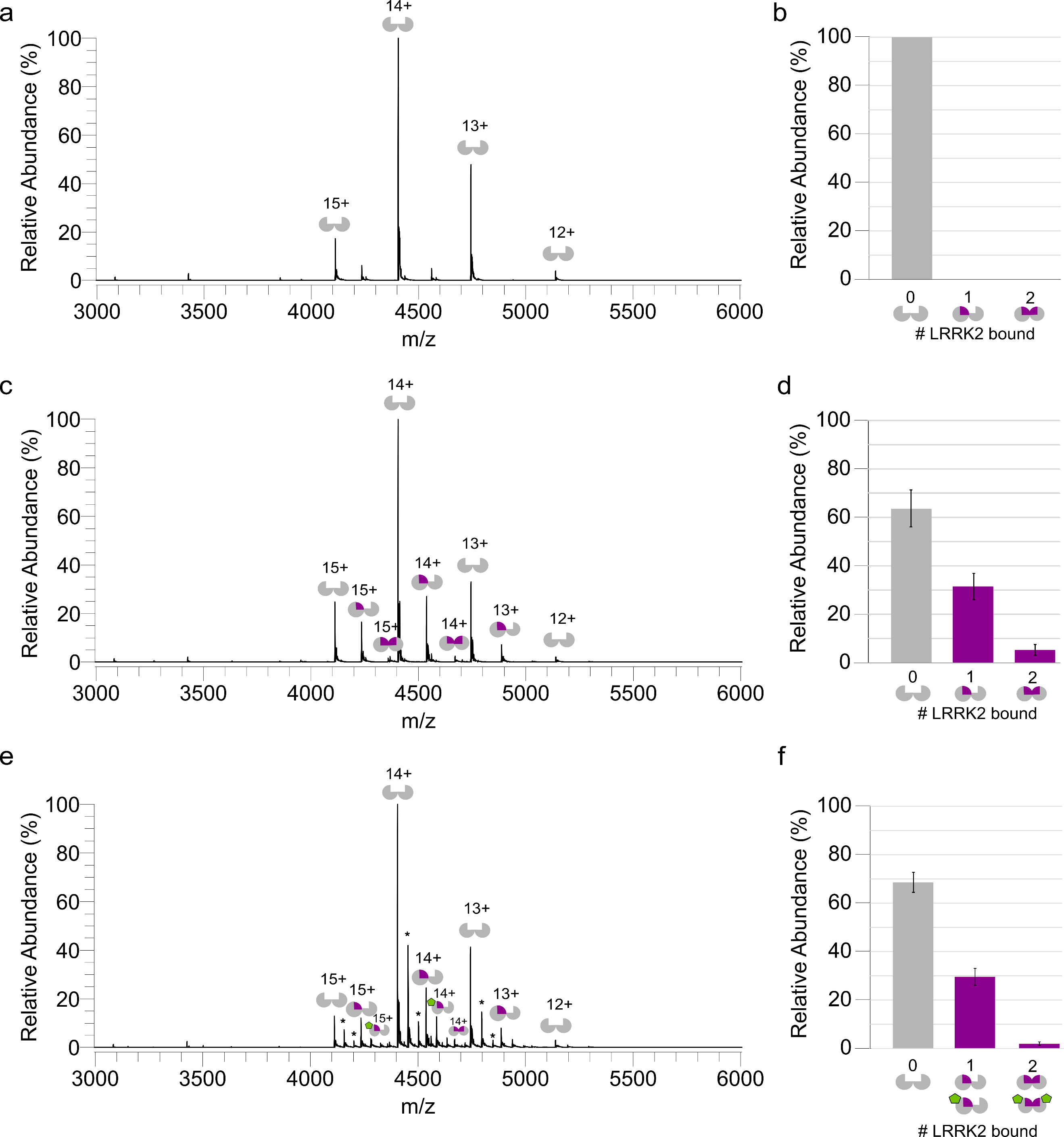
**

**Fig. S14. Native mass spectra showing that FC-A does not stabilize the interaction between 14-3-3σ and LRRK2.** Native mass spectra of 14:3:3 alone (a), 14:3:3 with LRRK2 in a 1:2 ratio (b), and 14:3:3 with LRRK2 and FC-A in a 1:2:10 14-3-3:LRRK2:FC-A ratio (c). The fraction of bound complexes observed are shown in b, d, and f, respectively. In the absence of FC-A, singly and doubly bound LRRK2 is observed in complex with 14-3-3. Upon addition of FC-A, unbound 14-3-3 still dominates the spectrum. * likely represents non-specific adducts of FC-A that form during the electrospray ionisation process due to the high concentrations of FC-A being used, however, low affinity interactions cannot be ruled out. The average relative abundance of each protein complex across 3 replicates is shown in the bar charts with the error reported as standard deviation.

**
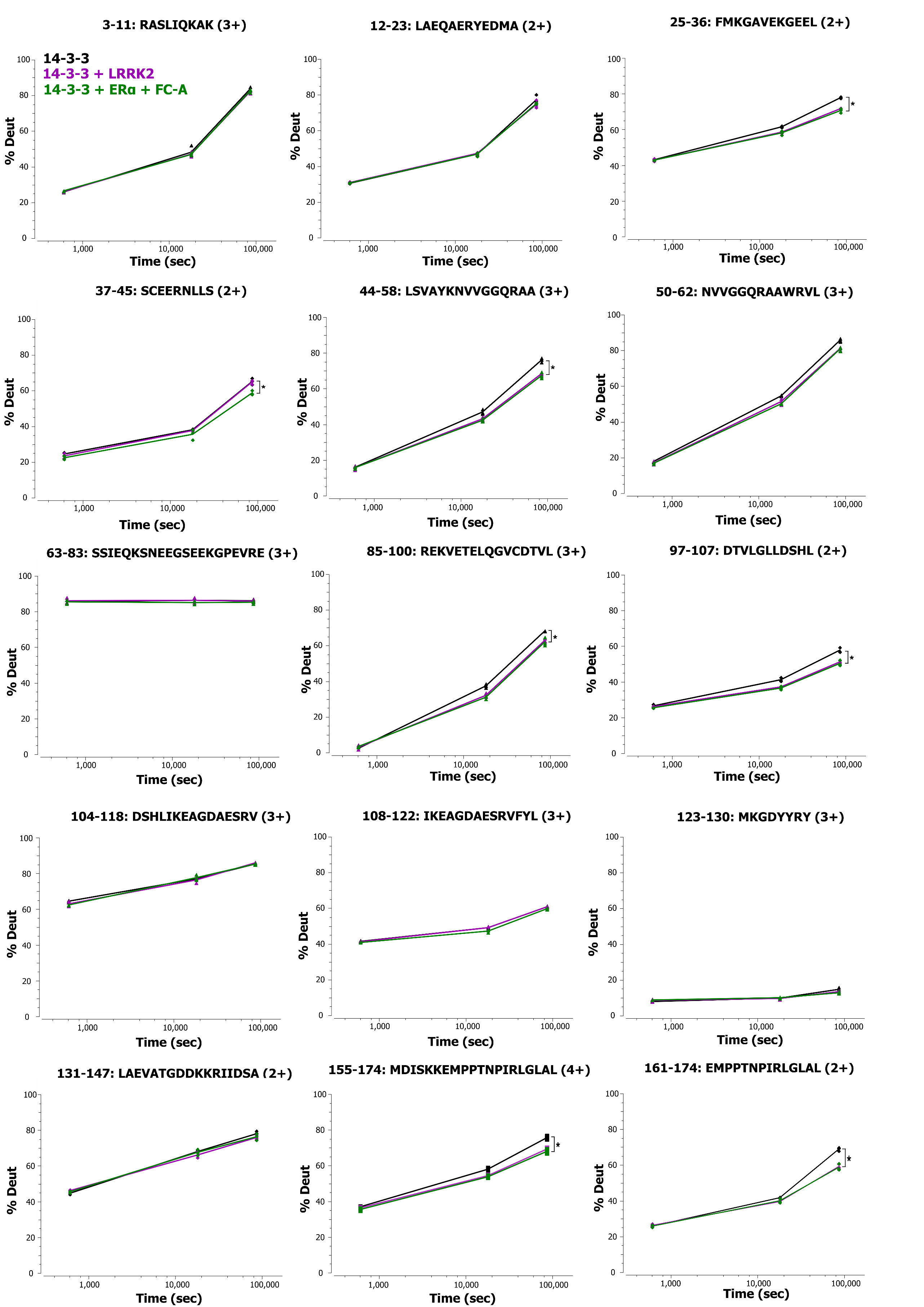
**

**
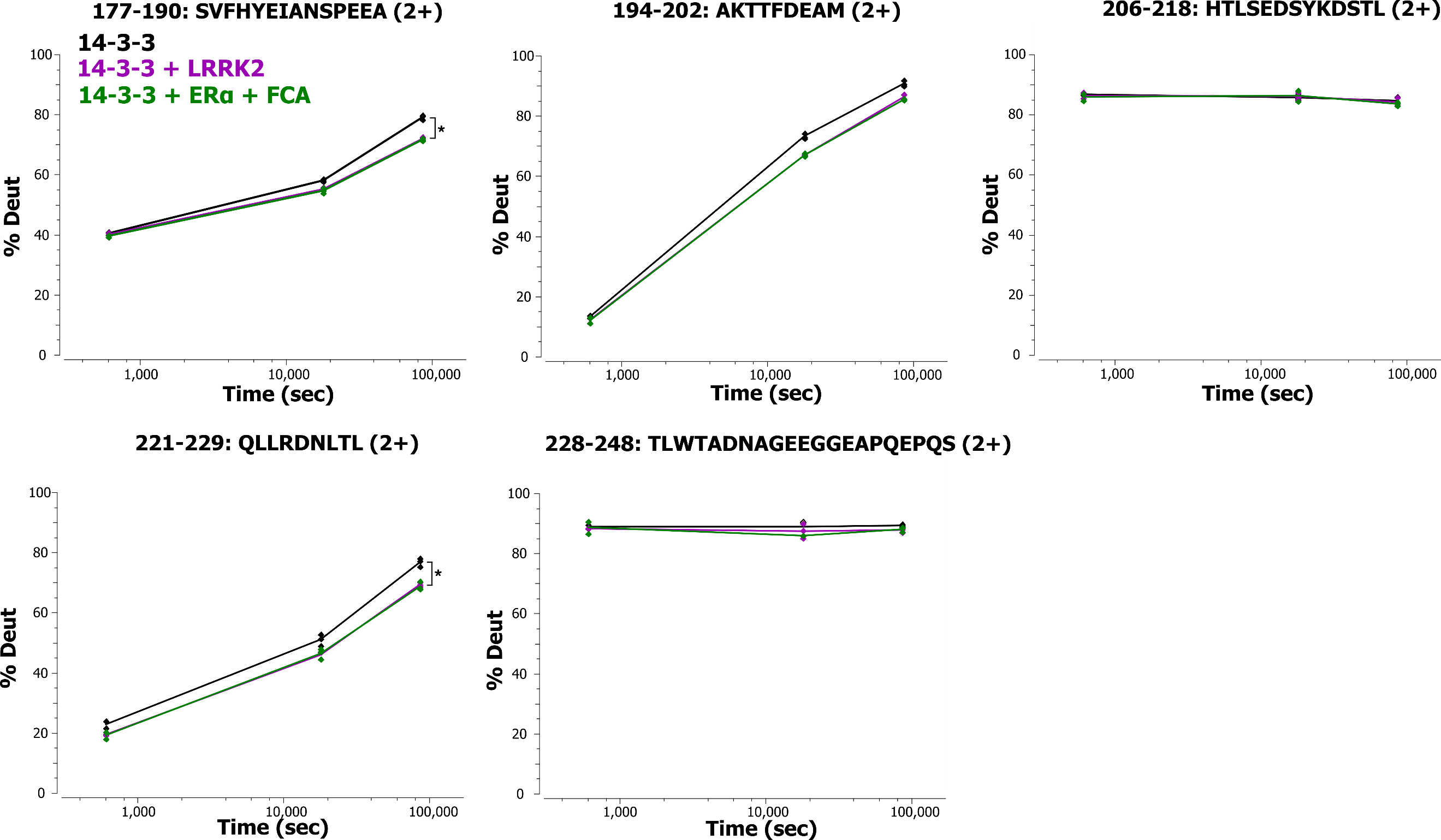
**

**Fig. S15. Uptake plots of peptides covering the 14-3-3 sequence displaying % deuterium (% Deut) incorporation at 10 minutes, 5 hours and 24 hours in the unbound (black),** **LRRK2 bound (purple) and FC-A bound (green) complex.** 20 out of 124 total peptides were selected to show deuterium uptake over time. The 20 peptides cover the majority of the 14-3-3 sequence. Peptide sequences and their residue numbers are shown above the plots with the corresponding charge state. * represents significant differences between protein complex conditions at 24 hours (p ≤ 0.05).

**
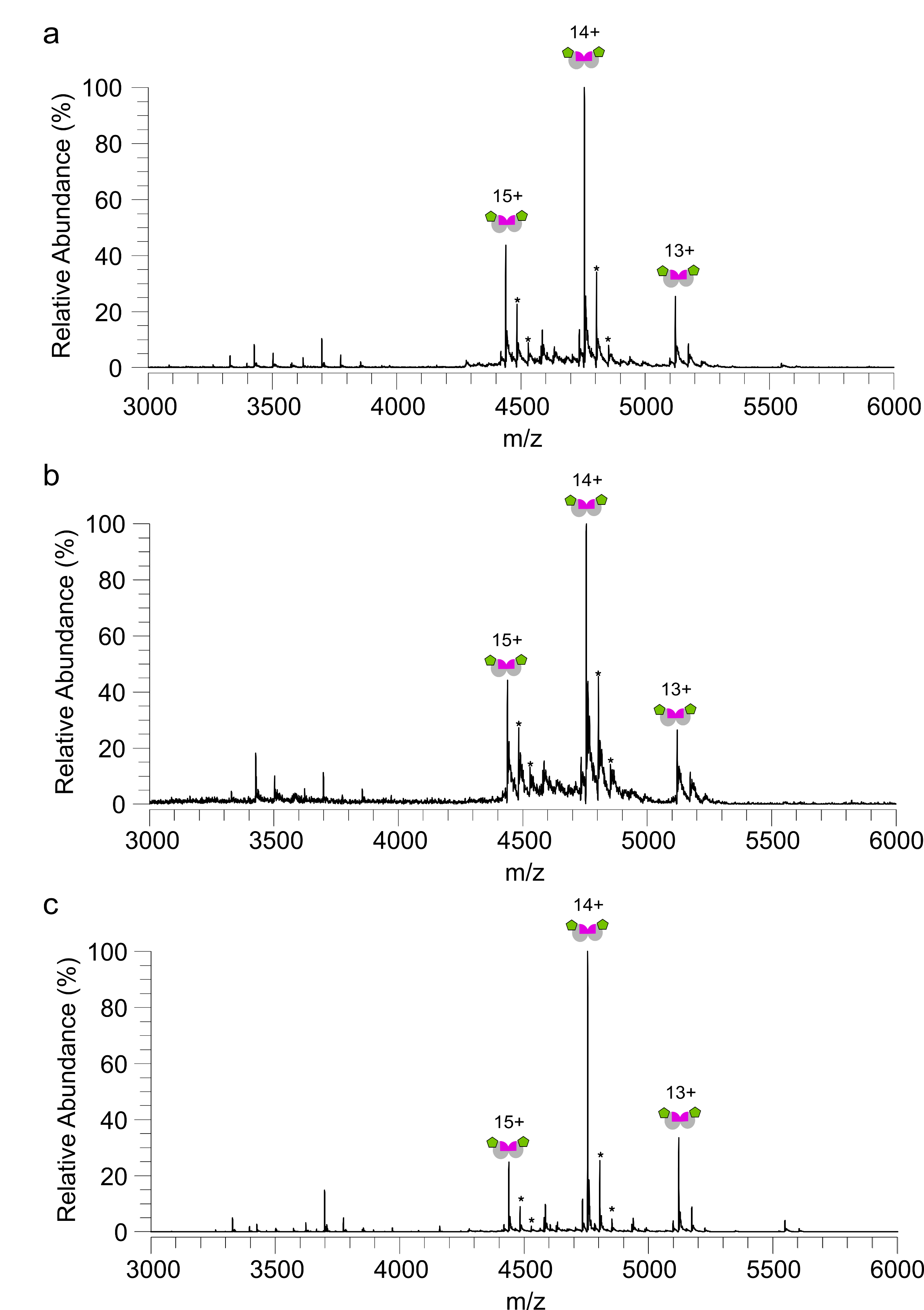
**

**Fig. S16. Native mass spectra of 14-3-3σ/ERα/FC-A conditions at 10min (a), 5h (b) and 24 h (c) to mimic conditions of HDX-MS experiments.** * likely represents non-specific FC-A adducts to the stabilized complex.

**Table S1. Theoretical and measured masses of protein complexes.** The theoretical mass accounts N-terminal methionine loss on 14-3-3. The error reported is the standard deviation measured between all charge states.

| Protein complex | Theoretical mass (Da) | Measured mass (Da) |
| --- | --- | --- |
| 14-3-3_2_ | 61664.5 | 61662.5 ± 0.03 |
| 14-3-3_2_**•**ERα | 63431.3 | 63430.2 ± 0.2 |
| 14-3-3_2_**•**ERα_2_ | 65198.1 | 65198.2 ± 0.2 |
| 14-3-3_2_**•**ERα_2_**•**FC-A_2_ | 66559.7 | 66560.0 ± 0.4 |
| 14-3-3_2_**•**LRRK2 | 63519.3 | 63518.2 ± 0.4 |
| 14-3-3_2_**•**LRRK2_2_ | 65374.2 | 65374.1 ± 0.1 |
| 14-3-3_2_**•**LRRK2**•**FC-A | 64200.2 | 64199.0 ± 0.5 |

Table S2. HDX-MS sample information

| Dataset | Apo 14-3-3σ | 14-3-3σ + ERα | 14-3-3σ + ERα + FC-A | Apo 14-3-3σ | 14-3-3σ + LRRK2 | 14-3-3σ + LRRK2 + FC-A |
| --- | --- | --- | --- | --- | --- | --- |
| HDX reaction details | Final D_2_O concentration = 90 % D_2_O in 100 mM ammonium acetate  P_D_ = 7.2  Temperature = room temperature  10 minutes, 5 hours and 24 hours  Maximally-labelled apo 14-3-3 control  29.6 % (22.6 % – 39.3 %) | | | | | |
| HDX time course |  |  |  |  |  |  |
| HDX controls |  |  |  |  |  |  |
| Back-exchange (mean and range) |  |  |  |  |  |  |
| Number of peptides | 124 | | | 110 | | |
| Sequence coverage | 96.4 % | | | 96.4 % | | |
| Average peptide redundancy | 5.4 | | | 5.0 | | |
| Technical replicates | 3 | 3 | 3 | 3 | 2/3 | 2/3 |
| Significant differences in HDX (delta HDX) | Apo vs ERα = 0.41 Da  95% confidence | ERα v FC-A = 0.33 Da  95% confidence | Apo vs FC-A = 0.44 Da  95% confidence | Apo v LRRK2 = 0.65 Da  95% confidence | LRRK2 v FC-A = 0.68 Da  95% confidence | Apo vs FC-A = 0.66 Da  95% confidence |

**Table S3. Raw HDX-MS data for apo 14-3-3σ, 14-3-3σ/ERα and 14-3-3σ/ERα/FC-A.** The values presented are averages across three replicates.

**References**

[1] M. Noike, Y. Ono, Y. Araki, R. Tanio, Y. Higuchi, H. Nitta, Y. Hamano, T. Toyomasu, T. Sassa, N. Kato, T. Dairi. Molecular Breeding of a Fungus Producing a Precursor Diterpene Suitable for Semi-Synthesis by Dissection of the Biosynthetic Machinery. *PLOS ONE* *7*, (2012), e42090.
